# Acetylcholinesterase Inhibitors in the Treatment of Neurodegenerative Diseases and the Role of Acetylcholinesterase in their Pathogenesis

**DOI:** 10.3390/ijms22179290

**Published:** 2021-08-27

**Authors:** Łucja Justyna Walczak-Nowicka, Mariola Herbet

**Affiliations:** Chair and Department of Toxicology, Faculty of Pharmacy, Medical University of Lublin, Jaczewskiego 8bStreet, 20-090 Lublin, Poland; lucja.wn18@gmail.com

**Keywords:** acetylcholinesterase, acetylcholinesterase inhibitors, neurodegenerative diseases

## Abstract

Acetylcholinesterase (AChE) plays an important role in the pathogenesis of neurodegenerative diseases by influencing the inflammatory response, apoptosis, oxidative stress and aggregation of pathological proteins. There is a search for new compounds that can prevent the occurrence of neurodegenerative diseases and slow down their course. The aim of this review is to present the role of AChE in the pathomechanism of neurodegenerative diseases. In addition, this review aims to reveal the benefits of using AChE inhibitors to treat these diseases. The selected new AChE inhibitors were also assessed in terms of their potential use in the described disease entities. Designing and searching for new drugs targeting AChE may in the future allow the discovery of therapies that will be effective in the treatment of neurodegenerative diseases.

## 1. Introduction

According to the International Union of Biochemistry and Molecular Biology (IUBMB), AChE belongs to the group of hydrolases acting on ester bonds of esters of carboxylic acids (3.1.1.7) [1].

The AChE molecule has an ellipsoidal shape with dimensions of approx. 45 × 60 × 65 Ǻ. It consists of 537 amino acids. The monomer of this enzyme is an α/β protein consisting of 12-stranded central mixed β sheet surrounded by 14 α-helices. The binding site of AChE consists of several sub-sites—catalytic (CAT), acyl pocket, oxyanion hole, anionic site and peripheral anionic site (PAS). The enzyme structure has a deep and narrow gorge, which penetrates halfway into the enzyme (length 20 Ǻ), extending toward the base of the enzyme. The base and lining of the gorge is composed of residues Asn-66 and Ile-444. In addition, it contains residues such as Asp-285, Glu-273, Asp-72, Tyr-334 and Glu-199 [2,3]. The residue of Glu-199 is associated with stabilization of the transition state, and the residue of Asp-72 is involved in trapping the ligand within the gorge [4]. In the gorge, nearly centrosymmetrically from each catalytic subunit, the active center is located at a distance of 4 Å from its base. It is also important that the active center has an arrangement of hydrogen bond donors, which allows stabilization of tetrahedral enzyme–substrate complexes. The catalytic triad of enzymes consists of serine (Ser-203), histidine (His-447) and glutamate (Glu-334) [2,5]. The aromatic groups in the active site play an important role in the binding of quaternary ligandins. The anionic site contains Trp-86, Trp-84 and Phe-330 residues [6]. The Trp-86 residue binds the quaternary ammonium group of choline and active center inhibitors, e.g., edrophonium through cation–pi interaction. The Phe-295 and Phe-297 residues are responsible for the substrate specificity of the acyl pocket for covalent adducts. The Phe-295 residue is also responsible for substrate specificity in the non-covalent enzyme substrate complex [7]. The oxyanion hole is constructed from the N-H groups of the backbone peptides, viz., Gly-121, Gly-122 and Ala-204. Residues Gly-121, Gly-122 and Gly-120 are part of a flexible “glycine loop”. The loop is adjacent to S203 and it is one of the gorge walls of the enzyme. This position ensures its contact with most non-covalent AChE ligands [5]. The amino acid Trp-286 present in PAS as well as the previously mentioned Trp-86 are important in the mechanism of inhibition by peripheral anionic site ligand, thus providing plasticity to the AChE active site [7]. As for the function of PAS, it is considered that it may act as an initial binding site for substrate entry to the acylation site or that it may modulate cation clearance and product release [4]. The “back door opening mechanism” is used to remove the reaction products from the AChE active site. The Tyr-442 and Trp-84 residues are responsible for it [8]. AChE can occur in a monomer, dimer or tetrameric form. For example, the tetrameric form (G4) is predominant in the brain and the dimeric form (G2) is found mainly on erythrocytes [3].

Neurodegenerative diseases are often associated with the cholinergic system, in which the main neurotransmitter is ACh, broken down by AChE. AChE inhibitors or muscarinic and nicotinic receptor (nAChR) agonists are often used to treat these diseases. Moreover, AChE has a number of different functions beyond the breakdown of ACh, including participation in inflammation, cell apoptosis, morphogenic and adhesion functions, as well as participation in oxidative stress. During neurodegenerative diseases and depressive disorders, inflammation, cell apoptosis and increased oxidative stress occur [9]. AChE also plays a role in the theory of catecholaminergic–cholinergic balance in depressive disorders [10].

AChE is present in several molecular forms [11]. The AChE-E form is found on red blood cell membranes and is associated with the Cartwright blood group (YT) [12,13]. The second molecular form of AChE is AChE-R. It is thought to play a role in oxidative stress and is also found in neurodegenerative diseases [14,15]. Its excess can also lead to disorders of spermatogenesis in rats and reduced sperm motility in humans [16]. In the nervous system, the AChE-S form is found in the highest levels [11]. One variant of AChE-S is N-AChE-S. It has an elongated N-terminus to which one of the exons gives rise. It is thought to be closely associated with apoptosis [15,17].

The enzyme has many different functions, including morphogenic function. AChE is responsible for the development of neuromuscular junctions, thalamocortical connections and axon growth during the nervous system development [18,19,20,21]. AChE may act as a mediator of cell architecture changes in neurogenesis and may also be responsible for neuroplastic processes in the neocortex [22,23].

AChE is also involved in cell apoptosis. Cells expressing AChE more readily undergo apoptosis, and inhibition of this enzyme also inhibits apoptosis [24,25,26,27,28,29]. It is also gaining interest that this enzyme is involved in the formation of the apoptosome. For the formation of the apoptosome, the necessary interaction is between AChE and caveolin and then with cytochrome c [30]. Silencing of the AChE gene resulted in inhibition of caspase 9 activation, interaction of activating factor protease 1 with cytochrome c and consequently, inhibition of apoptosis [31,32,33].

Due to the homology and structural similarity of AChE with cell adhesion molecules, the enzyme is presumed to have a role in cell adhesion [20,34]. An experiment was performed in which the level of substrate–cell adhesion correlated directly with the level of AChE expression. Adhesion was blocked by specific anti-ACHE antibodies or by the AChE inhibitor BW284c51 [35]. A similar experiment was also performed on fibroblasts and astrocytes, which confirmed the role of AChE in cell adhesion [36].

The aim of this review is to present the role of AChE in the pathogenesis of neurodegenerative diseases. Additionally, this review aims to evaluate the benefits of currently used AChE inhibitors in the treatment of these diseases. Selected novel AChE inhibitors were also considered for their potential applications in the described disease entities.

## 2. Acetylcholinesterase in Neurodegenerative Diseases

### 2.1. Alzheimer’s Disease

Alzheimer’s disease (AD) is one of the most common neurodegenerative diseases. The prevalence of this disease continues to rise. In the United States (US), it is estimated that the number of people with AD will reach 13.8 million by 2050. It is also projected that in 2025, the number of people 65 and older with AD will reach 7.1 million people, a 22% increase in incidence compared to 2020 [37,38]. In Poland, according to the 2016 Report of the Commissioner for Human Rights (RPO) on “The Situation of People with Alzheimer’s Disease”, it is estimated that more than 300,000 people suffer from the disease [39].

AD is characterized by progressive dementia and memory impairment. Additionally, neuropsychiatric symptoms appear over time and the patient becomes less and less independent. The diagnosis of this disease is always probable, as a definitive diagnosis can be made at autopsy. It is not completely known. Several hypotheses have been developed to try to explain its pathogenesis [40,41].

Due to the fact that in this disease, the cholinergic system is disordered, the so-called cholinergic hypothesis was developed. According to its proponents, even in the early stages of AD, cholinergic innervation may be disordered. Ch4 neurons are particularly susceptible to this degeneration. It is generally assumed that cholinergic function can be improved by AChE inhibitors or nicotinic or muscarinic receptor agonists. Both of these strategies are used in the treatment of AD patients. Overactivity of AChE leads to a decrease in ACh concentration, which in turn causes degeneration of the cholinergic system. The use of enzyme inhibitors can improve the life of a patient with AD, but the therapy is only symptomatic, i.e., it delays the symptoms’ onset and is not a complete cure [41,42,43]. The value of AChE activity determinations in the early stages of AD is quite limited, as only a mild decrease in the enzyme activity is observed. Significant changes in the activity do not develop until late stages of this disease [44,45]. AChE is present in the primary cleft, as well as in the postjunctional fold, but its largest fraction is associated with the basal lamina. In the primary cleft, AChE is found closer to the muscle surface than to the presynaptic membrane, whereas in the postjunctional fold, it is found along its entire length, reaching its maximum density at halfway down the fold [46]. Studies have shown that in early AD, the changes have a presynaptic character [47]. This would be consistent with the previous studies finding that AChE activity decreases only slightly in early disease [44]. In AD, neuronal apoptosis develops over time. AChE may also contribute to this. It has been shown that cells with increased expression of this enzyme on their surface undergo apoptosis more easily. In other studies on cell cultures, N-AChE-S was transfected. This resulted in activation of the Tau Glycogen synthase kinase 3 (GSK3), induction of Tau protein hyperphosphorylation and apoptosis [17,28].

Another hypothesis that attempts to explain the pathogenesis of AD is the amyloid hypothesis. Its proponents point to the accumulation of amyloid deposits in the brain as the cause of the disease. It is even supposed that the accumulation of these deposits precedes neurodegeneration and the symptoms’ onset [39,48,49,50]. β-amyloid (βA) has a neurotoxic effect on mature neurons, leading to their death [51]. βA is formed by proteolysis (endosomal/lysosomal/at the surface of the plasma membrane) of amyloid precursor protein (APP). This reaction occurs with the involvement of γ-secretase, a component of which is preselin 1 (PS1) [52,53,54]. This process is dependent on AChE. Modulation of PS1 by AChE occurs through a non-cholinergic pathway (at the protein level and at the transcript level). It is important to note that AChE inhibitors do not exert long-term beneficial effects on APP [55,56]. However, APP is also an important modulator of AChE at the transcriptional level and exerts repressive effects on this enzyme [57].

It has also been shown that AChE can participate in βA accumulation. The cortical activity of the enzyme is mainly associated with the amyloid core, diffuse preamyloid deposits and cerebral blood vessels. In AD, AChE presents differential sensitivities to inhibitors. Inhibition by indoleamine and bacitracin is a feature of AChE found in AD [58,59].

In addition, this enzyme can also interact directly with βA. The βA–AChE complex is more neurotoxic than βA aggregates alone. The molecular form that is responsible for binding to βA is the synaptic form of AChE (hAChE-S). It enhances βA fibrillization in vitro [60,61,62].

By conducting studies in transgenic mice, it has been shown that the h-AChE-S complex with βA becomes bound in the α-helix or β-sheet conformation. The β-harmonic form may be able to spread βA and is highly amyloidogenic [62]. AChE promotes and accelerates βA deposition, but the active center of the enzyme is not involved, and so this is a process independent of the catalytic properties of the enzyme [63,64].

βA tends to interact with the hydrophobic environment. Hydrophobic interactions play a role in the formation of the AChE–βA complex. AChE bound to plaques and tangles exhibits different properties than AChE bound to axons and cell bodies. The interaction of AChE with βA can induce small structural changes in the AChE molecule. PAS are presumed to play a role in amyloid formation. It is likely that it contains a hydrophobic environment that is favorable for the formation of the AChE–βA complex. Additionally, it is thought that the adhesion functions of AChE may be related to PAS [65].

In addition to PAS, the N-terminal region of AChE may also contribute to βA deposition. The β-hairpin region of AChE7-20 may cause βA aggregation. It has high structural similarity to the β-hairpin structure domain of βA [66]. The effect of hAChE degradation by insulysin (IDE) has also been studied and it has been shown that proteolysis of the non-amyloidogenic domain of hAChE changes its conformation into β-hairpins while releasing peptides that assemble into amyloid protofibrils [67].

Using Pittsburgh compound B (PiB), a positive correlation between AChE activity and PiB binding was observed. The researchers speculated that this may be related to the cholinergic anti-inflammatory pathway. The inflammatory process is an inherent feature of exacerbating AD. Thus, βA aggregates would induce an inflammatory response. The inflammatory effect associated with the previously mentioned pathway is that there would be an increased release of ACh into peripheral neurons [68,69,70].

As already mentioned, AChE in the brain occurs as a globular G4 tetramer. As AD progresses, there is a reduction or loss of the G4 form in favor of the G1 monomer in the cortex and cerebrospinal fluid (CSF). The increase in G1 form correlates with the density of amyloid deposits in the cerebral cortex [62,71].

In addition to the cholinergic and amyloid hypotheses, the tau hypothesis is gaining more attention. Proponents of this hypothesis believe that neurofibrillary tangles (NFTs), which are formed from hyperphosphorylated tau protein (p-tau), are the cause of AD. This protein is associated with microtubules. Its native state lacks a defined three-dimensional structure, making it highly prone to abnormal folding [41,72]. It was shown that overexpression of p-tau would cause an increase in AChE activity in the brain, resulting in a depletion of ACh in the nervous system. It has also been suggested that p-tau is required for changes in AChE expression, since the wild-type tau did not cause any changes in enzyme expression. The fact is that p-tau colocalizes with AChE mainly in cytoplasmic regions in cells [73,74,75].

### 2.2. Parkinson’s Disease

Parkinson’s disease (PD) belongs to the neurodegenerative diseases. It is the second most common neurodegenerative disease, after AD. Aggregates of Lewy bodies (LB) and neurites are thought to be the cause of this disease. They are deposited in the substantia nigra (SN) and lead progressively to the degeneration of the dopaminergic system, through neuronal degradation. It is estimated that symptoms appear when about 50% of them atrophy. The cholinergic system is also dysfunctional in PD: atrophy of the nucleus basalis of Meynert, cognitive impairment and dementia. Cholinergic deficits are more pronounced in PD than in AD [76,77,78,79].

During PD, there is a significant decrease in AChE activity. The decrease in enzyme activity is probably related to cholinergic degeneration. The decrease in its activity has been independent of motor function as well as disease severity. Higher impairment in AChE activity is observed in patients with dementia [80,81]. Low AChE levels in the cerebral cortex of PD patients without dementia are strongly associated with cognitive impairment, which correlates simultaneously with cholinergic degeneration. However, this correlation is variable. Approximately one-third of the patients demonstrated a reduction in the number of cholinergic endings. The variety of manifest symptoms in PD is due to the involvement of different parts of the brain [82,83,84]. In addition, men are more susceptible to cholinergic denervation of the neocortex than women. However, such differences are not found for thalamic denervation [85].

It has been reported that the early accumulation of α-synuclein in cholinergic neurons in the basal forebrain coincides with the occurrence of LB and neuronal loss in the SN. AChE activity was also observed to be lower in patients with early PD dementia, particularly in the cerebellar medial occipital cortex. This area is where the greatest cholinergic denervation occurs [86,87,88]. Cholinergic denervation contributes to depressive symptoms in PD. However, it is more evident when the patient additionally develops dementia [89].

βA deposits also play an important role in the pathophysiology of PD [90,91]. As mentioned previously, AChE may play an important role in βA deposition in the brain. It is possible that it may also enhance βA aggregation in PD.

In PD, there is postural instability and gait difficulty motor subtype (PIGD). This subtype of PD is characterized by low sensitivity to dopaminergic drugs. PIGD is one of the factors in the development of dementia. There is often an accumulation of βA in the brain in this subtype, which exacerbates cognitive impairment as well as those associated with PIGD [92]. It has also been shown that βA deposition in PD patients can independently aggravate apathy. There was a significant correlation between βA binding and apathy in these patients [93]. βA could be deposited in the cerebral cortex as well as in the striatum [94]. Gait dysfunction in PD is associated with cholinergic deficits of the basal forebrain and a higher risk of cognitive decline in PD. Patients with cholinergic and dopaminergic degeneration showed correlations with gait speed. In addition, cortical AChE activity was below normal in some patients [95,96]. Dysfunction of the pedunculopontine nucleus is associated with the impaired postural control and gait disturbances. Decreased cholinergic innervation of the thalamus and consequently decreased AChE activity is associated with increased postural sway [97,98].

Hyposmia, as well as disorders of rapid eye movements, occur in prodromal PD. Studies have revealed that olfactory dysfunction in PD is caused more by cholinergic denervation of the limbic system than by dopaminergic denervation [99]. In addition, abnormal rapid eye movement in patients with PD may be associated with cholinergic denervation [100]. A positive correlation between olfactory dysfunction and AChE activity has been proven [101,102].

Deposition of p-tau also occurs in PD. Deposits of this protein have been observed in the olfactory bulb in up to 80% of patients with PD. Its accumulation is probably connected with cognitive impairment and the development of dementia in people with idiopathic PD [103,104,105]. As mentioned previously, AChE promotes the accumulation of p-tau in the brain.

It is important to observe that AChE plays an important role in eye diseases. The treatment of this enzyme’s inhibitors had a positive effect on retina growth [33,106,107]. In PD, visual disturbances occur, with causes ranging from the retina to higher cortical areas of the brain. A dopamine deficiency is thought to be mainly responsible for the retina changes [108]. However, it is not excluded that AChE may also play a role in the pathogenesis of ophthalmic changes in PD.

A study was performed in which salivary AChE activity was examined in PD patients [109]. In addition to this, salivary flow and total protein concentration were measured. Patients with PD showed a decreased salivary flow, increased AChE activity and total protein concentration. In add-on, the AChE/total protein ratio was also increased. Statistical significance tests were also performed and indicated that the increase in AChE activity would not be associated with the increase in total protein. Patients in a more advanced stage of PD presented higher salivary AChE activities than patients in the early stage. However, AChE activity did not correlate with other motor disorders or impaired taste, smell, etc.

Various mutations can influence the progression and onset of PD, including mutations in the *LRRK2* gene and *DJ-1*. Mutation in the *LRRK2* gene is common in inherited Parkinsonism. It is inherited in an autosomal dominant pattern. In clinical practice, this variant of the disease is not distinguished from idiopathic PD. *LRRK2* has a role in inflammation. AChE activity in carriers of this mutation was compared with AChE activity in patients with idiopathic PD [110,111]. AChE activity was demonstrated to be significantly higher in carriers of mutations in the *LRRK2* gene. This is consistent with people with this mutation showing a slower disease course and therefore less severe non-motor symptoms. It has been noted that increased AChE activity may be associated with increased neurotransmission at cholinergic synapses of the thalamus and cerebellar cortex.

Mutation in the *DJ-1* gene is one of the mutations that promote PD. Mutation in this gene correlates with motor disorders related to motor coordination [112]. Mice with this mutation showed increased AChE activities in the central nervous system (CNS).

It is a fact that oxidative stress plays a significant role in the pathogenesis of PD. Its main source is the activation of glial cells [113]. As mentioned before, the probable form responsible for oxidative stress is AChE-R. Stress results is an increase in AChE caused by an increase in the expression of this form. Astrocytes are mainly responsible for the increase in AChE-R [114]. It is suggested that an increase in AChE-R expression may play an important role in the pathogenesis of PD. In addition, the increase in AChE-R may contribute to the degeneration of the dopaminergic system, due to its protective function against cholinergic effects [115]. It has been proven that any alteration in alternative splicing may be responsible for the development of PD. Mutations in the region encoding AChE as well as concomitant changes in the gene encoding paraoxonase contribute to the increased frequency of PD after insecticide treatment [116].

As mentioned in the previous chapter, AChE is involved in the process of apoptosis. This enzyme is involved in neuronal death by apoptosis in PD [117]. PC12 model cells for PD and SNpc in a mouse model showed an increase in AChE expression. Deficiency of the enzyme decreased apoptosis of dopaminergic neurons.

### 2.3. Huntington’s Disease

Huntington’s disease is also one of the neurodegenerative diseases. It is inherited in an autosomal dominant pattern. It is caused by a mutation in the *IT15* gene, which is located on the short arm of chromosome 4. This gene is responsible for a protein called huntingtin (HTT). HTT accumulates in cells leading to cell degeneration and finally death. As a result of the mutation, there is an increase in the number of CAG repeats, resulting in an elongation of the glutamine chain (polyQ > 36) near its anionic terminal. The age of development of the disease is inversely proportional to the number of repeats. The disease leads to cachexia and death after about 15–20 years. Symptoms include characteristic chorea movements, cognitive impairment and mood disorders [78,118,119].

It is worth pointing out that cholinergic neurons do not degenerate completely in HD, which has been confirmed in many studies. However, cholinergic dysfunction has been shown to develop in HD [120,121,122,123,124].

It has also been observed that in HD, there is not a death of cholinergic neurons, but a reduction in the expression of genes and proteins in this system. By performing studies on mice models in HD (R6/1), it was evidenced that AChE activity was reduced and consequently they developed cognitive deficits in the middle stage of the disease [125,126]. Furthermore, it has been suggested that the decrease in AChE activity may be a compensatory mechanism, as choline acetyltransferase (ChAT) and vesicular Ach transporter (VAChT) activities also decrease in HD.

The highest AChE activity in HD patients has been reported in the caudate nucleus, which is mainly responsible for functions related to motor processes [127].

3-Nitropropionic acid (3-NPA) is a compound used to cause brain changes in animals similar to the brain changes seen in HD. It is a mitochondrial poison. After its administration, oxidative stress, free radical release and the striatal degeneration and phenotypic abnormalities typical of HD occur [128]. In this study, injection of this compound was followed by increased AChE activity in the striatum, cerebral cortex and hippocampus. Subsequently, rats were treated with 17β-estradiol and genistein and a decrease in enzyme activity was observed. Cognitive abilities in rats improved after administration of these two compounds. In another study, the administration of 3-NPA was followed by activation of astrocytes (A1) and consequently increased oxidative stress [129]. In addition, there has been an increase in proinflammatory cytokines, which may exacerbate HD.

Microglia activation is observed in mutation carriers and could be detected up to 15 years before the predicted age of onset of HD. Furthermore, activation of these cells correlates with dysfunction of the striatal neurons [130,131]. Microglia may activate A1 astrocytes by secreting proinflammatory cytokines [132,133].

As mentioned previously, similar activation of glial cells also plays an important role in PD. It is possible that oxidative stress may lead to the release of AChE-R by reactive astrocytes also in HD. The increased form of AChE-R contributes to dopaminergic degeneration. In HD, degeneration within the dopaminergic system also occurs [126].

Furthermore, it has been shown that degeneration of the thalamostriatal may contribute in some way to dystonia in HD. It was also suggested that the cholinergic system may be involved in dystonia [134]. Cholinergic transmission has been repeatedly shown to be impaired in HD [125,126,135,136]. However, it was postulated that treatment with AChE inhibitors is not indicated in HD [137,138].

### 2.4. Multiple Sclerosis

Multiple sclerosis (SM) is one of the most common inflammatory and demyelinating diseases of the CNS. According to the Polish Multiple Sclerosis Society (PTSR), the number of people with SM in Poland is estimated at about 45–50 thousand. Annually, there are about 1300–2100 new cases in this country according to PTSR [139]. The incidence of this disease in Europe is 108/100ths of inhabitants. Interestingly, the disease has a geographical distribution, as the frequency of incidence increases with increasing latitude. The highest prevalence is between the ages of 20 and 40 years. Women tend to develop SM twice as often as men [140]. In SM, there are multiple, diffuse autoimmune inflammatory changes leading to myelin and oligodendrocyte damage. The inflammatory cells infiltrates contain mainly T-lymphocytes (mainly CD8+). Neurotransmission is disturbed. Axons in the early stages of the disease are preserved, but over time, they become irreversibly damaged. Inflammatory changes occur mainly in the white matter, but also in the brainstem, cerebellum, spinal cord and optic nerve and are dynamic in character. During SM, remyelination also occurs at all stages of the disease, but mainly in the inactive phase. The disease is characterized by a progressive course, usually with exacerbations (called relapses) and remissions [78,141,142].

As mentioned previously, AChE is a component of the “cholinergic anti-inflammatory pathway”. This enzyme exacerbates inflammation and stimulates the production of pro-inflammatory cytokines. AChE is also present on immune cells such as T lymphocytes, B lymphocytes, macrophages and dendritic cells [143].

**Figure 1:**
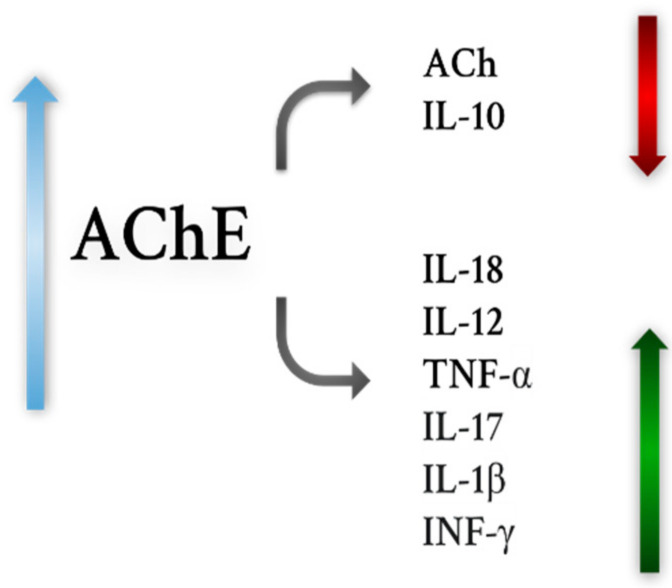
Associations between AChE activity and levels of pro-inflammatory cytokines, ACh levels and anti-inflammatory cytokines.

One hypothesis for the etiology of SM is based on the role of inflammation, as described above. Proinflammatory cytokines are increased during SM. It was observed that AChE activity is increased in patients with this disease in comparison to controls [144]. Moreover, AChE genotype rs2571598 and BChE rs1803274 were found to be more frequent in patients suffering from SM. Esterase activity is associated with ongoing inflammation and ACh levels. Some researchers postulate that peripheral AChE activity is a secondary marker to assess the role of the non-neuronal cholinergic system in regulating inflammation. Decreased ACh levels correlated with an increase in pro-inflammatory cytokines such as IL-17 and IL1-β in patients with the relapsing–remitting form of SM (RR-SM) in their CSF and serum [145]. A significant correlation was demonstrated between increased AChE activity and decreased ACh levels in RR-SM patients [146,147]. In addition, AChE transcript expression increased. The level of the enzyme increased more than 60% in RR-SM patients compared to the control group. Its G4 form was responsible for the increased serum AChE activity. In patients with SM, all components necessary for ACh synthesis and release were not changed, indicating that cholinesterases would be responsible for its decreased levels. Furthermore, higher AChE activity was associated with high levels of IL-18, IL-12/IL-23p40 and TNFα (pro-inflammatory cytokines). It has also been suggested that inflammation in the myelin sheath causes cholinergic dysfunction, consequently contributing to SM (Figure 1). Dysregulated ACh metabolism may be an additional pathological mechanism in SM, because levels of this neurotransmitter would affect cytokine levels [148].

An imbalance of cholinergic activity in the hippocampus of patients with SM has also been observed. This is consistent with some people with this disease disorder developing a variety of cognitive impairments. Decreased levels of ACh, decreased ChAT activity, but unaltered AChE activity were observed in the hippocampus of the patients studied. Overactivity of the enzyme in relation to the neurotransmitter that breaks it down may be the cause of these disorders [149]. Another study also observed unchanged AChE activity compared to the control group [150]. However, people with SM already had significant cognitive impairment. The researchers suggested that the unchanged activity may be connected to an increase in glial AChE, which offsets the decrease in neuronal AChE. According to these researchers, this would explain the inverse correlation between the activity of this enzyme and neuropsychological test scores, which would reflect a more pronounced glial response in patients with greater cognitive deficits. Furthermore, during the remission phase, cholinergic homeostasis between ChAT and AChE is established [151]. During this phase of SM, there is an increase in ChAT and a decrease in AChE. In the acute phase of the disease, there is a reversal of this balance, i.e., an increase in AChE and a decrease in ChAT.

There has also been an experiment conducted on Wistar rats by giving them ethidium bromide (the changes induced by this compound cause demyelination in the CNS) [152]. The rats were then treated with vitamin E and ebselen, two compounds well known for their anti-inflammatory and antioxidant properties. This study showed that these two compounds had an inhibitory effect on AChE in the cerebral cortex and hippocampus. These regions are responsible for learning and memory. Cognitive impairment occurs in SM. Demyelinating changes were less in the group in which these compounds were administered. The researchers suggest that this neuroprotection may be related to the antioxidant properties of both compounds. Thus, it seems likely that inhibition of AChE may be an important strategy in the treatment of SM, not only by increasing Ach availability, but also by regulating the “cholinergic anti-inflammatory pathway”. A microRNA (miRNA) is also involved in this pathway; more specifically, it is miR-132 targeting AChE [69,153]. It enhances the anti-inflammatory response. Its inhibition increased pro-inflammatory mediators and exacerbated SM in an animal model. This confirms the significant role of the enzyme in the pathogenesis of SM. SM patients also have reduced manganese levels in PMR compared to controls [154,155]. Moreover, high concentrations of this element have an inhibitory effect on AChE [156]. It may be speculated that there is a correlation between AChE activity in SM and manganese concentration.

### 2.5. Amyotrophic Lateral Sclerosis

Amyotrophic lateral sclerosis (ALS) is a neurodegenerative disease. It involves degeneration in the corticospinal tracts, brainstem and anterior horn cells. This leads to motor and non-motor symptoms. The cause of this disease is unknown. The disease has a heterogeneous character, i.e., the phenotype of symptoms, pathogenesis and genetic predisposition are variable. Therefore, it is postulated that the more correct term is syndrome rather than disease entity. The peak incidence is between the ages of 50 and 75. After this age, the risk of ALS decreases. There are two main forms of ALS: sporadic (SALS) and familial (FALS). Postmortem in ALS patients as well as in mice, neuronal degeneration was paralleled by an inflammatory response involving astrocytes and microglia proliferation. A characteristic feature of ALS is the presence of aggregates of the dysfunctional protein TDP-43. This protein normally localizes to the cell nucleus. Abnormal folding of TDP-43 causes deposition of its aggregates in the cytosol, leading to loss of nuclear function and dysregulated transcription. The disease has a localized onset, but then spreads [157,158,159,160].

The important thing is that disorders of the glutamatergic system play an important role in ALS pathology [161]. The cholinergic system is also affected in ALS. Both systems are related to each other. The possible involvement of AChE in ALS pathology was suggested already in the 1990s [162]. Release of this enzyme from motor neurons after stimulation of glutamine receptors or blockade of receptors for glycine preceded all detectable neuropathological changes. The presence of AChE on astrocytes caused their activation and motor neuron defects, as well as macrophage infiltration in the brain. This enzyme was released as a result of motor neuron overactivity. Another study confirmed that extracellular excess of glycine increases AChE secretion in vivo and in vitro [163]. The excess enzyme blocked cholinergic synapses, consequently leading to cholinergic dysfunction, which is well known to occur in ALS.

Furthermore, inhibition of AChE expression improved survival and also delayed the onset of ALS and inhibited motor neuron loss in transgenic mice (G93A-SOD1) [164]. The gene encoding AChE demonstrates reduced expression in proximal regions of spinal segments innervating the site of symptoms in ALS [165]. As previously mentioned, in ALS there is a deposition of the abnormally folded protein TDP-43. In Danio reiro with knockdown of the gene for TDP-43, symptoms similar to those found in ALS were induced [166]. Working on this animal model, an important interaction between AChE and TDP-43 was demonstrated. This protein can regulate AChE levels. Loss of TDP-43 function resulted in decreased AChE expression. Overexpression of the enzyme reduced phenotypic traits (neuromuscular junction defects, motor signs) in Danio rerio. Confirmation of these associations is provided by a study showing increased ACh release (3.5 times) in Danio reiro with TDP-43 depletion [167].

In the progress of ALS, inflammatory response is present. This disease leads to an increase in proinflammatory cytokines such as IL-1 (IL-1α, IL-1β), TNF-α, IL-6 and INF-γ [168,169]. The role of AChE in the regulation of inflammation has been repeatedly mentioned in this review. Therefore, it is likely that it may also play an important role in ALS. Reactive astrocytes and microglia cells are also found in ALS. In ALS, there is also excessive oxidative stress [170]. R-AChE plays a significant role in oxidative stress, but reactive astrocytes may be its source. It is possible that this molecular form of AChE contributes to the pathogenesis and pathophysiology of ALS.

### 2.6. Olivopontocerebellar Atrophy, Spinocerebellar Ataxia and Progressive Supranuclear Palsy

Olivopontocerebellar atrophy (OPCA) is not a single disease entity. During its course, it results in progressive cerebellar ataxia with spastic paresis of the lower limbs and dysarthria. There are several types of this disease entity. As for the sporadic form, the most common type is the type with multiple system atrophy (MSA) [171]. AChE activity was evaluated in seven patients with familial OPCA and compared with the enzyme activity values in patients with other neurodegenerative diseases such as AD and PD. Reduced activity of the enzyme was observed in the cerebellum and hippocampus in patients with OPCA. However, the greatest reduction in the activity was in the cerebral cortex (greater than or equal to that in PD and AD) [172]. AChE activity was also studied in different types of spinocerebellar ataxia (SCA) and cerebellar variant of multiple-system atrophy (MSA-C) [173]. In these studies, decreased AChE activity was observed in the thalamus of patients with SCA-3 and MSA-C, and in the cerebellar cortex of patients with MSA-C.

Another neurodegenerative condition in which AChE is somehow associated with its pathology is progressive supranuclear palsy (PSP). The disease manifests with PD symptoms, cognitive impairment, visual palsy and subcortical dementia. The cause of this disease is identified in the accumulation of p-tau, and therefore, it belongs to tauopathy [174]. Cholinergic degeneration also develops in this disease. In the CSF of patients with PSP, a reduction of one-third in AChE activity was observed compared to controls [175]. In PSP, the development of inflammation as well as increased oxidative stress and activation of microglia occur [174,176]. Moreover, microglia play an important role in the pathogenesis of this disease. The role of AChE in oxidative stress, inflammation and its association with microglia activation is mentioned many times in this review. It is possible that AChE may contribute to the pathogenesis and development of this disease. Moreover, PSP belongs to the tauopathies. AChE enhances the accumulation of p-tau. It is highly likely that in PSP, AChE may play a significant role.

### 2.7. Depressive Disorders

**Figure 2:**
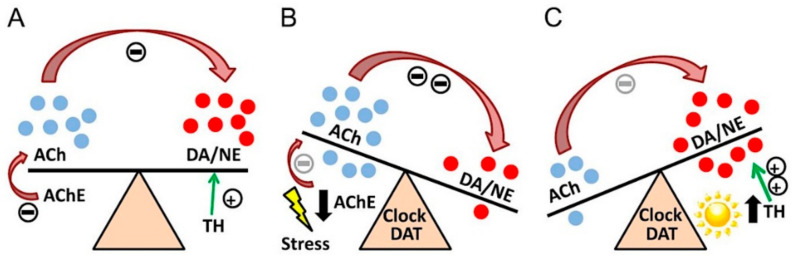
Theory of catecholaminergic–cholinergic balance in physiology, BPAD. (**A**) normal; (**B**) Depression; (**C**) Mania; [10]. Image obtained with permission license number: 5136601386126.

According to a 2017 WHO report, the total number of people suffering from depressive disorders worldwide is estimated around 322 million. The disease is more common in women than men [177]. As for Poland, it has been estimated that in 2017, there were 1 million people with depression and 288,000 with bipolar disorder [178]. Depressive disorders include major depressive disorder (MDD) and bipolar affective disorder (BPAD). The diseases are diagnosed using the ICD-10 classification system and the American DSM-5 classification. Between 10–15% of people diagnosed with major depression of a severity requiring hospitalization commit suicide. Typical symptoms of MDD include prolonged depressed mood, impaired emotional perception, feelings of exhaustion and loss of energy (the latter of which is only mentioned in the ICD-10 classification). The presence of one of two or three symptoms is necessary for the diagnosis of MDD. In addition, there are also symptoms connected with mental processes such as disorders of the train of thought, disorders of the content of thinking and suicidal tendencies and symptoms connected with somatic processes such as sleep disorders, disorders of motor activity and disorders of appetite and body weight. A necessary condition for the diagnosis of MDD is the duration of symptoms (at least 14 days). The most commonly used drugs are serotonin reuptake inhibitors (SSRIs). Monoamine oxidase inhibitors, drugs affecting noradrenergic, serotonergic and dopaminergic transmission, are also used in therapy. In some cases, drug-resistant depression may develop (nowadays increasingly) [179,180]. Dysfunction in the cholinergic system may be responsible for cognitive symptoms in MDD, especially in drug-resistant and long-term depression [181]. BPAD is characterized by alternating episodes of mania and depression. Symptoms of mania include irritability, hyperactivity, racing thoughts, psychotic symptoms and feelings of grandiosity. One theory regarding its development is the catecholaminergic–cholinergic balance theory. An increase in ACh activity may cause a decrease in dopamine activity and thus an increase in depressive symptoms. During mania, it has been suggested that this relationship may be reversed, i.e., a decrease in ACh activity will cause an increase in dopamine (Figure 2) [10,182].

Cholinergic interneurons in the nucleus accumbens are the main site of action for the serotonin care protein p11, which is responsible for depression-like mood changes. Researchers consider these interneurons to be responsible for anhedonia and behavioral despair in rodents. These interneurons are also the main source of ACh in the nucleus accumbens [183,184]. Elevated ACh levels in the CNS may contribute to depressive symptoms [185]. Moreover, in lipopolysaccharides (LPS)-induced depression, decreased AChE activity was demonstrated and it was suggested that these changes may be involved in depression complications [186]. The following studies showed that AChE inhibitors and muscarinic receptor agonists caused changes in the expression of biomarkers associated with mood disorders: increases in adrenocorticotropic hormone, β-endorphin, cortisol and lack of cortisol suppression after dexomethasone stimulation [187,188]. They also caused disturbances in sleep phases. Administration of physostigmine to rats increased their anhedonia and anxiety. Blood cell AChE was evaluated in 24 patients with mild MDD [189]. The enzyme activity was higher in patients with mild MDD compared with controls. The researchers suggested that the elevated enzyme activity was related to oxidative stress in this disease. They also consider that the increase in AChE was a compensatory mechanism for the high ACh levels in these patients. In contrast, another study in bulbectomized mice observed an increase in AChE in the hippocampus, but not in the prefrontal cortex [190]. The researchers suggest that it is AChE activity that influences the reduction of ACh in the hippocampus, and consequently, the onset of depressive symptoms in these animals. However, they consider that the procedure of removing the olfactory bulb may be connected with some induced changes in the enzyme activity.

Oxidative stress may be associated with depressive disorders. AChE activity was found to be increased in PC12 cells as a result of oxidative stress [191]. Furthermore, it was shown that O_2_•− may be the nodes that modulate the enzyme activity and the occurrence of depression-like symptoms. It is possible that a form of AChE-R, which is known for its role in oxidative stress, may be responsible for the increase in AChE. A group of adolescents (*n* = 310) in agricultural regions of Ecuador were also screened for AChE activity [192]. It was observed that inhibition of the enzyme by organophosphate pesticides during the agricultural season was associated with increased depressive symptoms in adolescents. Thus, it was shown that inhibition of AChE can affect mood. A similar study also examined a group of 529 adolescents in agricultural regions and found an association between decreased AChE activity and the onset of depression in adolescents, particularly in adolescent girls [193]. Furthermore, adults exposed to organophosphate pesticides were also examined [194]. In this study, patients who attempted suicide were chronically exposed to organophosphate pesticides and demonstrated reduced blood cell AChE activity. In addition, chronic exposure to these compounds may be one of the determinants of attempted suicide.

ACh levels can also be changed by stress, which increases the secretion of ACh. Administration of fluoxetine (a drug used for treatment of MDD) increases AChE activity especially in the hippocampus and thus leads to a decrease in ACh and restoration of cholinergic balance [195]. However, stress can also cause an increase in AChE activity as a compensatory mechanism, thus reducing ACh levels. As is well known, stress is one of the factors that promote the onset of MDD. Mice overexpressing AChE-R exhibited chronic stress-related behavior, which induced an increase in anxiety. Furthermore, exposure to stress increases AChE-R expression [196]. Long-term nicotine administration had an anti-anxiety effect in mice with AChE-R overexpression, but had no effect in control mice. Furthermore, AChE-R overexpression was associated with increased expression in anxiety-related genes, i.e., BDNF’s proteolytic activator MBTP1, BDNF’s receptor and BDNF’s coactivator of mitogen-activated protein kinase, PTPN11. Overexpression of these proteins is associated with an increase in the brain-derived neurotrophic factor (BDNF), which in turn enhances anxiety-related activities. In another study, Wistar rats exposed to chronic stress for 21 days demonstrated reduced AChE activity in the hippocampus [197]. During acute stress, mice demonstrated a decrease in AChE activity in the detergent-soluble fraction (G4), whereas under chronic stress, they showed a decrease in AChE activity in the salt-soluble fraction (G1) [198].

As mentioned earlier, sleep phases are disturbed in the course of MDD. There is a decrease in NREM, a decrease in REM latency and an increase in REM density [199]. The role of AChE is worth noting from this perspective. REM phase deprivation causes an increase in AChE activity and, consequently, a decrease in ACh [200,201]. There is a higher release of ACh during REM phase compared to the NREM phase [202], thus confirming the involvement of the cholinergic system in the course of MDD.

Mice were examined for the involvement of the cholinergic system in the pathophysiology of MDD [203]. Social defeat stress (SDS) model was used for this study. Animals in the study group were treated with physostigmine. Behavioral analysis was performed using open field test (OFT), social interaction test, sucrose preference test (SPT), elevated plus maze test and forced swim test (FST), and body weight was measured. Reduced AChE activity by its inhibition by phizostigmine resulted in increased cortisol levels in the animals. Animals treated with physostigmine and the SDS model showed decreased social interaction and increased depression-related behaviors. Animals that were only exposed to SDS showed no significant differences in AChE activity. Changes in the cholinergic system may cause different responses to stress.

In the development of MDD, there is a dysfunction of the immune system. Rats were injected with LPS and PC12 cells were also studied in vitro to examine whether ketamine produces an antidepressant effect through activation of the cholinergic anti-inflammatory pathway [204]. Ketamine showed an effective antidepressant effect by acting on α7 nAChR. In MDD, there is an increase in pro-inflammatory cytokines such as IL-6 and IL-8 [205]. The level of AChE activity in CSF was only non-significantly lower in MDD patients compared to control group. In MDD, lower levels of AChE in CSF were associated with higher plasma IL-6 levels but lower markers of microglia activation in CSF. However, a limitation of this study is that blood cell AChE, which is responsible for the regulation of the cholinergic anti-inflammatory pathway, was not determined. However, it was reported that the cholinergic anti-inflammatory pathway is up-regulated in MDD. Fluoxetine (an SSRI inhibitor) was administered to mice and it was demonstrated to affect cholinergic tone by altering serotonin levels and consequently increasing AChE activity [206]. Furthermore, an increase in AChE activity correlated with a decrease in anxiety behaviors in mice in OFT.

## 3. AChE Inhibitors in the Treatment of Neurodegenerative Diseases

### 3.1. Rivastigmine

Rivastigmine belongs to the carbamate inhibitors that inhibit AChE and BChE (Table 1). Rivastigmine forms a complex with esterases via a covalent bond, resulting in their temporary inactivation. It reacts to the anionic and ester sites of AChE. This results in a general increase in ACh. Indication for oral administration of rivastigmine is the symptomatic treatment of mild to intermediate dementia in patients with AD and idiopathic PD. Adverse effects include gastrointestinal disturbances, weight loss, increased extrapyramidal symptoms, allergic skin reactions, psychiatric disorders, cardiac arrhythmias and vascular disorders. Rivastigmine is characterized by dose-dependent efficacy. It can be administered orally or as a transdermal patch. This compound is found in the literature under two nomenclatures: SDZ ENA 713 and ENA 713 [207,208,209].

The drug (at a dose of 6 to 12 mg/day orally or 9.5 mg/day in the form of a transdermal patch) could permanently improve cognitive function and the ability to carry out daily activities in patients with AD. Moreover, rivastigmine reduced disease progression in various clinical trials. Patients demonstrated improved cognitive abilities in all tests used to assess cognitive abilities [208,210]. AD patients who had taken the drug for 5 years demonstrated significantly lower cognitive decline compared to the predicted decline if they had not been treated [211]. Although rivastigmine is recommended for intermediate AD, it has also been proven effective in severe AD, but in the form of a transdermal patch. It also improved cognitive ability in these patients. Since this drug has dose-dependent efficacy, the dose in the patch is 13.3 mg/24 h [208,212,213]. Orally administered rivastigmine requires a high level of dose control due to side effects. Because of this, transdermal patches of rivastigmine are becoming more popular. This drug easily penetrates through the skin. This eliminates the need to daily swallow the drug, which is difficult in AD due to the fact that these patients tend to forget. In addition, transdermal administration of rivastigmine helps to increase appetite in AD patients. It is also common for AD patients to have difficulty swallowing, so the transdermal patch seems to be a better option in them than the oral form. The drug in this form has fewer side effects. One of them is itching of the skin, as well as erythema [214,215,216]. In addition, rivastigmine reduces βA levels, has neuroprotective effects and modifies the APP processing pathway toward the non-amyloidogenic pathway. It also changes the unfavorable ratio of AChE-R to AChE-S in the CNS [208,217,218,219,220]. AD patients with vascular risk factors may demonstrate an altered response to rivastigmine treatment compared to AD patients without these risks (cognition and speech are particularly affected) [221].

As mentioned before, rivastigmine is approved to treat forms of dementia in people with PD. It has beneficial effects on cognitive abilities and also improves executive function in PD [222,223,224]. The drug has been proven to increase the left frontal lobe activity in PD patients, and as a result, patients showed improved ability to focus attention and improved executive functions [225]. However, the study negates the improvement in overall cognitive ability after administration of this drug. Moreover, rivastigmine reduced neuropsychiatric disorders, i.e., sleep disturbances, hallucinations and anxiety in PD patients with dementia who were maintained on anti-Parkinsonism drugs [226,227]. The drug also had a beneficial effect in reducing depression in these patients. In contrast, other researchers suggest that the use of rivastigmine to treat apathy in PD with dementia has limited effect [228]. They observed patients for 12 months and the drug did not result in significant improvement. There was only a slight reduction in symptoms. In PD patients without dementia, 6 months of drug administration resulted in a reduction in apathy, but the patients’ quality of life did not significantly improve [229]. The researchers suggested that this result may have been due to a partial reduction in apathy or that PD patients are not always aware of apathy symptoms. Orthostatic hypertension is very common in PD patients with dementia. This then impacts disease progression and the onset of cognitive impairment. Patients with this hypertension in PD benefit more from treatment with rivastigmine [230]. They showed a greater degree of cognitive improvement than patients without this hypertension after treatment with this drug. An increased frequency of falls occurs in PD. Patients were monitored for 12 months and the drug reduced the frequency of falls [231].

The effect of rivastigmine on cognitive impairment present in HD has also been studied [232]. However, the results of the studies are quite controversial. In one of them, memory, learning ability and executive functions were evaluated. There were no statistically significant differences in the group where patients were treated with rivastigmine in improving cognitive function. In contrast, in another study, patients were administered rivastigmine regularly for 2 years [233]. Various scales such as Total Functional Capacity (TFC), Mini-Mental State Examination (MMSE), Marsden and Quinn Chorea Severity Scale, as well as Abnormal Involuntary Movement Scale (AIMS) were used to assess cognitive function. Based on these scale scores, patients treated with rivastigmine showed significant improvements in their cognitive and executive abilities, and there were also improvements in their motor skills. Other researchers have observed patients for 8 months [234]. After that time, they reported only slight improvements in cognitive and motor skills. However, the patients in the study demonstrated a trend toward improvement in these abilities, as well as a reduction in choreic movements. Rats (Wistar) were administered 3-NPA and then treated with rivastigmine [235]. Administration of rivastigmine was observed to improve locomotor ability and grip strength. In addition, the rats demonstrated improved memory. Rivastigmine reduced the oxidative stress induced by the administration of 3-NPA.

The use of rivastigmine has also been tried in SM. In this disease, there are changes in the processing speed [236]. The effect of a single dose of the drug was studied and it improved the processing speed and also the activation of bilateral frontal regions was more extensive. The opposite results were obtained in a study in which patients received rivastigmine for 16 weeks [237]. Only a non-significant increase in recall ability was shown, but not at the level of statistical significance. In contrast, another study administered rivastigmine to patients for 12 weeks [238]. They were assessed using the Wechsler Memory Scale (WMS) at the beginning and end of treatment. As with the previous study, there were no statistically significant differences for those taking rivastigmine versus placebo in terms of mean total memory score of WMS. However, the drug did produce small but significant improvements in memory and significant differences in some subscales of the WMS. Short-latency afferent inhibition (SAI) was also evaluated in patients with RR-SM and secondary progressive SM (SP-SM) after a single dose of the drug [239]. After administration of rivastigmine, there was an improvement in SAI, and consequently, an improvement in verbal memory. In a mouse model of SM, rivastigmine reduced the degree of demyelination and decreased the inflammatory infiltrate caused by T lymphocytes and microglia cells [240]. However, the drug dose was not effective when clinical symptoms occurred. The researchers hypothesized that this was because rivastigmine inhibits the immune response peripherally and does not directly affect neurons and oligodendrocytes. Rivastigmine also had immunomodulatory effects on T lymphocytes.

As for SCA-3 and the use of rivastigmine, it proved to be ineffective in improving gait in these patients. However, some of the patients who took the drug longer showed improvement in limb coordination [241].

PSP patients with dementia were administered rivastigmine for 3–6 months [242]. Only one patient out of five reported severe nausea. The drug had beneficial effects on memory function, fluency of speech and working memory after only 3 months. However, all patients experienced deterioration in motor skills. The researchers suggest that these latter changes may be related to disease progression.

### 3.2. Donepezil

Donepezil is a selective reversible AChE inhibitor. Its indication is for the symptomatic treatment of mild, moderate and severe dementia in AD. Side effects are similar to those of rivastigmine and mainly involve gastrointestinal disturbances, but muscle cramps and insomnia may also occur [243,244]. This drug has a relatively long half-life. Its undoubted advantage is that it interacts only slightly with other drugs and that a meal consumed by the patient does not affect the pharmacokinetics of the drug [245]. Donepezil can be currently administered in three types of doses: 5 mg, 10 mg and 23 mg. Low doses of donepezil viz: 5 mg and 10 mg inhibit cortical AChE activity only up to 20–40%. In patients with more severe AD, a higher dose has been approved because in these patients the cholinergic deficits are greater [246,247]. Higher doses of donepezil were not associated with increased side effects in patients who took it for about 1 year [248]. In addition, the 23 mg dose is in the form of a matrix type tablet. Therefore, it is not immediately released [249]. This drug is also characterized by a dose-response relationship [247,250]. Donepezil improves cognitive function in AD and is associated with spontaneous activity in the brain: right gyrus rectus, right precentral gyrus and left superior temporal gyrus [251]. In addition to its effects on cognitive ability, it also preserves function in people with severe AD. It alleviates some of the cholinergic deficits in such patients, but it does not extend survival time in these patients [252]. Moreover, the drug reduced hippocampal atrophy in patients with prodromal AD compared to the placebo group after one year of its administration [253]. Donepezil also reduces neuropsychiatric symptoms associated with AD [254]. Furthermore, the effect of CYP2D6 polymorphism was studied and demonstrated to affect the response to donepezil [255]. The G allele rs1080985 in the *CYP2D6* gene was associated with a worse response to treatment in patients with AD. Patients carrying the ApoE ε4 allele may respond better to treatment with this drug as tested in patients with mild to moderate AD [256]. However, the meta-analysis showed that this allele did not affect the response to donepezil [257]. The researchers speculated that a more likely theory is the presence of polymorphisms of this allele affecting this response. An interaction between p-tau and donepezil has also been demonstrated. This drug binds to this protein and more specifically to the R2 region, which is responsible for microtubule binding [258]. Therefore, the researchers suggested that donepezil could be a potential inhibitor of p-tau aggregation. Besides the interaction with p-tau, donepezil reduced serum βA levels in AD patients compared to controls. Moreover, it was associated with improved cognitive function in the study group [220,259].

The effect of donepezil on mild cognitive impairment was studied in PD [260]. After 48 weeks, the drug did not reduce these impairments, but improved patients’ electroencephalography (EEG) scores. The donepezil-treated group had higher levels of theta/beta2 (TB2R) bilaterally in the frontal, temporal and occipital cortex. In contrast, TB2R was lower in the control group, which was associated with the progression of the disease. Furthermore, donepezil was more effective in PD patients with dementia who demonstrate smaller decreases in AChE [261]. The effect of donepezil on the development of psychosis and cognitive impairment in patients with PD without dementia was studied. Patients received the drug for 2 years [262]. The initial dose of donepezil was 3 mg/24 h (2 weeks after the start of the study) and was then increased to 5 mg/24 h (4 weeks after the start of the study). The control group received a placebo. Cognitive function was assessed using the MMSE and the WMS and Frontal Assessment Battery (FAB), and sleepiness was assessed using the Epworth Sleepiness Scale (ESS). The drug did not prevent the development of psychosis in PD patients without dementia. In addition, donepezil had a beneficial effect on cognitive function in patients who had a higher MMSE. However, it did not perform well in the group with lower MMSE. The drug also improved the WMS and ESS scale scores. In contrast, there was no significant difference in FAB classification between the donepezil and placebo treatment groups. The researchers suggest that the drug may be effective in preventing cognitive decline but not in preventing psychosis in people with PD without dementia. In contrast, PD patients with dementia showed improvement in cognitive function and general condition after donepezil treatment [263]. It was also noted that the safety profile of donepezil was consistent with that of AChE inhibitors. In PD patients with dementia, increasing the dose of donepezil from 5 mg to 10 mg improved the mean MMSE score [264]. Continuous therapy with this drug did not affect receptor dysfunction (receptor desensitization). The drug also had a beneficial effect on reducing pattern hallucinations and improved the depression index. One case report of a PD patient who exhibited hypersexual behaviors in relation to the disease reported a reduction in these behaviors after donepezil administration [265]. Researchers suggest that donepezil may be beneficial especially when such behaviors are compulsive in nature without producing negative motor consequences.

As for SM, researchers also tried to test the use of donepezil in the treatment and alleviation of cognitive impairment. This drug improved memory function, attention, quality of life and depression rates in these patients [266,267,268]. Researchers suggested that starting donepezil therapy early in the stages of SM may help alleviate the intensity and slow the progression of cognitive impairment. In contrast, in another study, this drug did not improve memory or other cognitive impairment in SM patients after 24 weeks of treatment [269,270].

The effect of donepezil on cognitive deficits was also assessed in a mouse model of R6/2 HD [271]. It was shown that donepezil administered long-term can reduce cognitive deficits in a mouse model of HD. However, HD patients (two females and six males) had a high dropout rate from donepezil (50%) [272]. In addition, some patients experienced aggravation of chorea after taking the drug. Patients were assessed using the Unified Huntington’s Disease Rating Scale (UHDRS). Statistical significance was observed in the motor part of the UHDRS. In contrast, there was no statistical significance in specific subsets including dystonia, bradykinesia, visual impairment, chorea movements or rigidity. The researchers noted no improvement in cognitive, behavioral or motor scores after donepezil treatment in these patients. Another study only confirmed the fact that donepezil was not an effective drug in alleviating cognitive impairment, improving quality of life or reducing chorea movements [273].

Donepezil was also administered for 6 weeks to patients with PSP. Similar to rivastigmine, deterioration in motor function was also observed [274]. The drug only slightly improved memory in these patients.

### 3.3. Galantamine

Galantamine belongs to tertiary plant alkaloids originally isolated from bulbs of the *Amaryllidaceae* family. It belongs to selective inhibitors of AChE, and the inhibition of the enzyme by it depends on its dose. It exhibits a 53-fold stronger selectivity for action on AChE than BChE. This drug has a large volume of distribution. Side effects are similar to those of other AChE inhibitors. This drug is recommended for use in patients with mild to moderate forms of AD. It can be administered in three doses: 8 mg/24 h, 16 mg/24 h and 24 mg/24 h [275,276,277]. However, recent studies have shown that this drug does not affect nAChR as was commonly supposed. Researchers hypothesized that the beneficial effects of galantamine may be due not only to inhibition of AChE but also to other mechanisms, but this mechanism would not be the activation of nAChR [278]. Galantamine can be an extended-release or immediate-release medication. Both formulations induce improvements in cognitive function in patients with AD. They also show no significant differences in side effects among the two formulations, and they had similar effects on improving cognitive function. However, there was no evidence of improvement in the overall condition of AD patients after galantamine administration compared to the placebo group [279]. Galantamine demonstrated efficacy in improving cognitive function in patients with severe AD, but similar to the previously mentioned study, there was no improvement in the patients’ general status [280].

In a 36-month study, AD patients (194 patients) were treated with galantamine. In this experiment, continuous and long-term treatment with this drug was able to slow down the rate of cognitive decline [281]. Patients treated with galantamine maintained cognitive function at baseline for the first 12 months of therapy. The drug delayed cognitive decline by about 18 months compared to patients in a control group. Approx. 50% of patients demonstrated improved cognitive function compared to the predicted value in untreated patients. However, galantamine has the most beneficial effect on long-term memory [282]. In addition to cognitive function benefits, this drug significantly reduces mortality in patients with mild to moderate AD [283].

Furthermore, galantamine reduces LPS induced inflammation by decreasing the expression of astrocyte and microglia activation markers (CD11b and GFAP), pro-inflammatory cytokines (IL-1β, IL-6 and TNF-α). It also prevented an increase in inflammation in hippocampal neurons [284]. This drug also affects synaptic proteins including Synaptophysin (SYN). Reductions in presynaptic SYN and postsynaptic PSD-95 protein were demonstrated after treating mice with LPS. SYN and PSD-95 deficiencies correlate with cognitive decline in AD. Galantamine caused attenuation of the decline in these proteins. The drug also prevented cognitive deficits in mice, i.e., spatial learning and memory. Galantamine also counteracts the oxidative stress induced by βA, which promoted nerve cell survival and prevented autophagy [285]. The effect of galantamine on psychotic symptoms in dementia has also been studied [286]. The drug worked effectively as a first-line treatment in dementia patients who developed these symptoms. Because of its broad safety profile, the researchers suggest that such therapy is worth considering before including typical antipsychotics. The efficacy of long-term galantamine therapy in AD patients is likely to be related to the response to short-term therapy with this drug [287,288]. Furthermore, plasma galantamine concentrations are not significantly related to treatment efficacy [282,289].

Mice treated with galantamine demonstrated, besides benefits for cognitive impairment, reduced levels of βA in the brain and decreased glial cell activation [290]. It is probable that this drug binds to βA mainly by van der Waals forces with its central region (Lys16-Ala21) and C-terminal region (Ile31-Val36) [291]. This prevents the formation of toxic βA oligomers. In addition, this drug would act to promote the formation of non-toxic Aβ 1–40 oligomers. Galantamine was also tested in PD patients without dementia [292]. Patients took the drug for 16 weeks at different doses (from 8–24 mg/24 h). The dose was increased as the study progressed. The drug did not work effectively in these patients. There was no improvement in attention, memory, visuospatial performance or quality of patients’ lives. In addition, there was a high dropout rate due to side effects such as gastrointestinal disturbances and worsening of symptoms associated with PD. In contrast, another study reported positive effects on cognitive impairment on the MMSE and the Alzheimer’s Disease Assessment Scale-Cognitive Subscale (ADAS-cog scales) compared to baseline and control group in PD patients with dementia [293]. There were significant decreases in the severity of psychotic symptoms, behavioral and emotional disturbances. In addition, no symptoms of disease severity were observed. These results are confirmed by a study involving 16 PD patients with dementia [294]. There was observed improvement in cognitive abilities, motor skills and reduction in hallucinations (and even their complete disappearance).

In an animal model where 3-NPA was administered to induce HD, neurological improvement was demonstrated after galantamine administration [295]. The higher dose produced better effects, but the difference was not statistically significant. In addition, the group treated with galantamine demonstrated less neuronal loss compared to the control group. The size of the striatum changes was also reduced, which indicates that the striatum was clearly rebuilt after treatment. The drug also prevented apoptosis of the striatum. Furthermore, in one case report, a patient with HD and psychosis had improved motor skills as well as reduced psychotic symptoms after galantamine administration [296].

### 3.4. Huperzine A

Huperzine A is a plant alkaloid isolated from *Huperzia serrata.* It belongs to the reversible and selective inhibitors of AChE. It is not yet registered as a drug for any neurodegenerative disease (approved only in China for the treatment of mild to moderate AD). However, it is sold as a dietary supplement. This compound has a strong neuroprotective effect, so it was decided to use this property in the treatment of neurodegenerative diseases [297,298,299]. Huperzine A improved cognitive function and daily activities in AD patients [300,301]. A longer treatment duration would most likely result in better efficacy of the drug. In terms of side effects, there currently are not many studies regarding the safety profile of this compound. Only mild to moderate cholinergic side effects were reported. Patients with AD demonstrated significant improvements in cognitive function and improved ability to perform tasks [302]. Patients were treated with the drug at a dose of 0.2 mg twice daily for 8 weeks. The second phase study tested the dose dependence of Huperzine A and its effect on cognitive function [303]. Patients were evaluated on the ADAS-cog scales and the MMSE. A dose of 200 µg twice daily did not significantly improve cognitive abilities in patients with mild to moderate AD [304]. Significant effects were observed at a dose of 400 µg twice daily. In addition, the compound may have beneficial effects not only on cognitive function in AD, but also normalize the excitation-inhibition ratio in the cerebral cortex, and thus may have potential uses in the epilepsy that sometimes accompanies AD, as well as having disease-modifying effects on the course of the disease.

The compound does not only inhibit AChE, but also has a beneficial effect on βA deposition. Therefore, it reduces neurodegeneration and memory impairment. In addition, it changes the direction of APP processing towards the non-amyloidogenic α-secretase pathway and reduces oxidative stress induced by hydrogen peroxide (H2O2) by increasing the activity of antioxidant enzymes [297,299,305]. Because of these properties, it is possible that this drug may also be effective in other neurodegenerative diseases.

### 3.5. Phenserine and Its Enantiomer

Phenserine is a phenylcarbamate derivative, structurally resembling rivastigmine. It is a selective, non-competitive inhibitor of AChE. It does not interact with PAS [306]. In 1995, phenserine was proposed as a candidate for the treatment of AD [307,308,309]. The drug was well tolerated in animals and showed beneficial effects on AD development also in cell cultures. It improved cognitive function in animals and decreased APP and βA expression. It acted post-transcriptionally at the level of the 5′-untranslated region of AβPP mRNA. However, this drug has failed in clinical trials. Many researchers suggest that the failure was due to methodological mistakes.

This drug was tested on animals that developed traumatic brain injury (TBI), a disorder which is a significant risk factor for developing neurodegenerative diseases [310]. Increased inflammation occurs during the course of this disease. Phenserine attenuated inflammation by reducing the ratio of activated to resting microglia. In another study, phenserine showed neuroprotective effects in neuronal cell cultures, but also acted positively on spatial and visual memory in a mouse model of TBI. It also reduced oxidative stress and had a protective effect in cell cultures by protecting them from glutamate excitotoxicity [311]. It has been shown that phenserine reduced pro-inflammatory cytokines such as IL-1β, with no effect on IL-10 (an anti-inflammatory interleukin) in phytohemagglutinin-induced inflammation [312]. Furthermore, phenserine reduced cognitive deficits and prevented neurodegeneration in the hippocampus and lateral cortex in a mouse model of TBI [313]. Moreover, in the same study, the drug caused inhibition of microglia and astroglia activation in the hippocampus and cortex. Phenserine demonstrated inhibitory effects on TNF-α production and improved synaptic function and synapse plasticity [314]. Cells from suspected AD patients and healthy patients were also studied. It was observed that phenserine decreased IL-1β and TNF-α release in cells from AD patients and in phytohemagglutinin-treated cells. However, it did not affect the release of Il-10 (as confirmed by the aforementioned study).

The effect of phensernine was also examined in patients with mild AD. Patients underwent a PET scan [315]. The group receiving phenserine showed an increase in regional cerebral metabolic rates for glucose (rCMRglc). The researchers suggest that this increase may also be related to improved cholinergic function, because the functions of the cholinergic system are highly dependent on energy. However, no changes in mean cortical PiB retention were observed. The authors of the study justify this result by the fact that phenserine does not affect the APP already present, but reduces the production of new APP. There was also no effect of the drug on biomarkers in plasma or CSF. However, there was a tendency to increase β-amyloid 40 (Aβ40) (a possible protective role in AD) in plasma and CSF, which, together with rCMRglc, correlated with improved cognitive function.

Soman belongs to the organophosphorus structured war gases. Phenserine is lipophilic in character and therefore easily penetrates into the brain. It selectively inhibits AChE but without altering protein expression. Soman-treated rats showed neuroprotective effects of this drug and increased their survival; in addition, pretreatment improved their motor performance [316]. The researchers suggest that its beneficial effects are not only due to AChE inhibition, because posiphen, which is not among the AChE inhibitors, also showed improvement in this regard. It is thought to mitigate neuronal cell death. Phenserine showed a sustained but mild effect on AChE inhibition in CSF in AD patients [306]. Treatment with the drug reduced S-AChE in AD patients who had previously received donepezil.

In cell cultures, phenserin had a dose-dependent neuroprotective effect via PKC and MAPK (mitogen-activated protein kinase) pathways [317]. It also exerted beneficial effects on cells expressing APPSWE (APP variant associated with familial AD). Phenserine also showed a protective effect against H2O2-induced death. It is noteworthy that phenserine induced neuroprotective, anti-apoptotic effects in cellular and animal models of hypoxia [318]. The antiapoptotic effect of the drug may be related to the ERK-1/2 signaling pathway, and thus, it induced a decrease in caspase 3 activity, APP levels and the expression of matrix metallopeptidase-9 and glial fibrillary acidic protein (GFAP). Phenserine, on the other hand, increased BDNF and B-cell lymphoma 2 (Bcl-2) levels.

Phenserine has also been tried in the treatment of PD [319]. It showed a beneficial effect on lowering α-synuclein levels. However, it acted no differently than its enantiomer, i.e., interacting with other elements of the 5′UTR or with the 3′UTR. The researchers also noted that the RNA target in the 5′UTR of APP mRNA was similar to that in the α-synuclein transcript.

Posiphen, the enantiomer of phenserine, has also been tried for the treatment of AD. It is a much less effective AChE inhibitor than phenserine [310]. However, like its isomer, it has beneficial effects in reducing APP expression and βA. It was also tested in animals with TBI and showed advantages in relieving inflammation, but not as much as phenserine. In a rat model of soman poisoning, rats pre-treated with posiphen showed improved motor performance [316]. It was shown that the compound had a neuroprotective effect. In cell cultures and in mice, it reduced APP levels, presumably by downregulating α-secretase [320]. It showed a dose-dependent response. However, it did not affect β-secretase and γ-secretase. In another study, this compound was shown to be successfully distributed to the brain [321]. It also positively affected the levels of APP and its C-terminal fragments, CTFα and CTFβ. The compound also decreased soluble APPα and soluble APPβ as well as t-τ and p-τ in CSF [322]. In addition, the effects of posiphen and its major metabolites ((+)-N1-norPosiphen, (+)-N8-norPosiphen and (+)-N1, N8-bisnorPosiphen) on APP and α-synuclein formation were tested [323]. Inhibition of the production of these compounds was observed after treatment with posiphen and its metabolites. Its metabolites showed anticholinesterase activity without affecting nicotinic and muscarinic receptors. Posiphen exerted beneficial effects by affecting proinflammatory cells and IL-1β levels. It was also observed that posiphen induced a dose-dependent neurotrophic effect in cell cultures [317]. This compound, like its enantiomer, probably acted through PKC and MAPK signaling pathways. In addition, it induced more beneficial effects on cells expressing APPSWE. It also protected cells from the adverse effects of H2O2.

Posiphen, as well as phenserine, has also been tried in the treatment of PD. It has shown a beneficial effect towards inhibition of α-synuclein accumulation [319]. It was also noted that its primary metabolites also exert this effect. It had an effect by targeting the 5′UTR. Posiphen inhibits α-synuclein translation [324]. It is thought that it may be a good candidate for the treatment of PD because it does not have a strongly anticholinesterase effect. In addition to affecting α-synuclein translation, it normalized colonic motility which was associated with a decrease in pathological protein in the intestine and brain [325]. Clinical trials are currently underway for the use of this compound in the treatment of AD and PD [326,327].

## 4. The Use of AChE Inhibitors for MDD and BPAD

Most of the commonly used AChE inhibitors show a dose-dependent response, as previously mentioned. It is suggested that their use in the treatment of depression is also associated with a dose-dependent response [328]. Researchers suggested that these inhibitors induce antidepressant efficacy only at lower doses. The researchers consider these inhibitors to be Janus-faced or U-shaped in their antidepressant properties. They also suggest that appropriate dosing may provide a therapeutic benefit for patients with depressive symptoms. In addition, the researchers suggest that stress may have modulatory properties for the effects of AChE inhibitors.

Donepezil was tried in late-onset depression (LOD) as maintenance treatment [329]. A group of patients were given donepezil with an antidepressant. The drug only resulted in cognitive improvement in patients who had cognitive deficits. In patients without cognitive impairment, the researchers noted no benefit in use. However, donepezil administration increased the recurrence of depressive episodes. LOD is a known risk factor for dementia. A 78-year-old female patient responded well to 5 mg donepezil [330]. After 3 weeks, she had reduced depressive symptoms, reduced suicidal thoughts and improved mood, but no improvement in cognitive ability. In another case report of LOD, the patient was taking venlafaxine and donepezil [331]. The patient progressed into remission of depression and cognitive improvement occurred. In addition, the effects of AChE inhibitors in elderly patients with dementia on symptoms of depression and anxiety have been studied [332]. However, no relationship was established between the use of enzyme inhibitors and depression and anxiety in elderly patients with dementia. The lack of effect of donepezil on symptoms of LOD and cognitive impairment while patients were on antidepressant treatment is also confirmed by another study [333].

Drugs that affect sigma receptors may have beneficial antidepressant effects. Donepezil interacts with sigma receptors [334,335]. In mouse studies in FST, it was reported that the antidepressant effect of donepezil may be related to its interaction with these receptors [336]. However, in this experiment, the researchers suggest that donepezil should be administered at higher doses; this is due to the fact that for sigma 1 receptors, higher concentrations of agonists are required.

In another study, donepezil was administered at a dose of 10 mg and tested its effect on REM phase in patients with MDD [337]. Donepezil statistically significantly improved sleep latencies in depressed patients compared to the placebo, but did not affect the percentage of REM and NREM sleep.

In mouse studies in FST, donepezil demonstrated antidepressant properties. The study analyzed the effect of the dose of this drug on the antidepressant response [338]. Acute administration of the drug at low doses produced antidepressant-like effects in mice. The researchers also suggest that ACh has an inverted U-shaped relationship with mood. SSRIs used in patients with MDD often cause cognitive impairment in these patients. A study was conducted on 73 patients who were divided into two groups [339]. One group received a placebo and the other received donepezil. It was shown to improve cognitive abilities in patients taking SSRIs.

Galantamine, rivastigmine and physostigmine were also tested in mice. The mice were exposed to FST and OFT [340]. This study concluded that these drugs may be effective for treating MDD, but their effects are dose-dependent. At low doses, they may have an antidepressant effect, but at high doses, they may make depression worse (U-shaped dose response). Furthermore, a reduction in depressive symptoms has been demonstrated in AD patients after treatment with AChE inhibitors (donepezil or rivastigmine) [341]. Significantly, these changes were independent of changes in cognitive function. They were assessed at the beginning of the study and after 16 weeks on the Geriatric Depression Scale (GDS). Rivastigmine and donepezil were administered to 96 male Wistar rats. [342]. They were exposed to chronic stress. The rats were divided into two groups, one group received rivastigmine (2 mg/kg) and the other received donepezil (0.3 mg/kg). The drugs were administered for 5 weeks. Rivastigmine and donepezil reversed the effects of chronic stress. They demonstrated antidepressant, anti-anxiety and pro-cognitive effects. The researchers suggest that the controversial results of their study may be due to the fact that AChE inhibitors can interact with muscarinic and nAChR. These receptors have different contributions to the pathophysiology of depression. They also suggest that the beneficial effects of donepezil and rivastigmine may be due to their effects on nAChRs rather than muscarinic receptors.

Rivastigmine was administered to patients with AD who have MDD [343]. They were followed up for 6 months. The drug was administered in the form of a transdermal patch. It has been suggested that the drug in this form may improve the frequency and severity of depression in patients with mild AD. However, it is not known whether rivastigmine had an antidepressant effect because of its use in the treatment of AD. Thus, its effect may have been related to improving AD-related symptoms.

In another study, rivastigmine was administered to mice after removal of the olfactory bulb for 2 weeks [344]. The mice showed improvement in the OFT, locomotion and novelty-suppressed feeding test and tail suspension test (TST). The researchers suggest that the antidepressant effect of the drug may be related to its property of enhancing neurogenesis in the hippocampus. In addition, this study noted that rivastigmine acted on serotonergic 5HT1A receptors, which would be responsible for hippocampal neurogenesis.

Post-traumatic stress disorder (PTSD) and post-concussion syndrome (PCS) are also often accompanied by MDD [345]. Rivastigmine has been attempted as an addition to treatment in PTSD. In a series of case reports, rivastigmine had a beneficial effect in reducing symptoms of post-traumatic stress disorder [346]. However, a study on a larger group did not confirm this effect. In another study, patients with PTSD and PCS were treated with galantamine for 12 weeks [347]. Galantamine did not significantly affect cognitive ability, but improved episodic memory. In addition, in patients with PCS, the drug reduced depressive symptoms, while in patients with PTSD, it did not reduce any symptoms associated with the syndrome.

The effects of galantamine on LOD symptoms have also been studied [348]. However, no beneficial effect of this drug was observed, but the researchers suggest that increasing the dose could accelerate the antidepressant response in these patients. In addition, all patients received venlafaxine XR concurrently. There was also a high dropout rate due to side effects. In another study, patients received galantamine (8 mg for 4 weeks, then 16 mg for another 4 weeks) or a placebo and were additionally maintained on antidepressants. Galantamine had a beneficial effect on patients with higher Hamilton Depression Rating Scale (HDRS) scores [349]. In patients with lower HDRS, it demonstrated no improvement. Galantamine significantly improved mood and cognitive abilities in a patient with MDD in AD. The patient was only taking galantamine as monotherapy. The effect of galantamine on EEG recording in patients with MDD was also investigated [350]. The drug was demonstrated to decrease absolute brain power after 8 weeks of use compared to the placebo. This was visible especially for the beta waves; the researchers suggest that galantamine may reverse hypoactivation of the brain, and thus be an effective drug for treating MDD [351].

Huperzine A was tested in post-stroke depression in rats [352]. After 4 weeks of Huperzine A administration, there was an increase in SPT and a decrease in immobility time in FST, indicating an antidepressant effect of the drug. There was improvement in neurological and cognitive functions in these animals. In addition, there was an increase in the expression of 5HT1AR, pCREB and BDNF and an increase in dopamine, norepinephrine and 5-hydroxytryptamine after administration of Huperzine A. In a randomized meta-analysis, Huperzine A showed no significant improvement in depressive symptoms [353]. However, the group in which it was used as an adjunctive treatment showed significantly greater improvement in cognitive function than the group in which only the antidepressant was used.

In one case report, a patient was administered rivastigmine, donepezil and galantamine at various intervals as needed [354]. The patient was suffering from psychotic depression. After treatment with rivastigmine, the patient’s hallucinations resolved and there was an improvement in mood. However, he developed salivation. He was then treated with donepezil; this did not have a beneficial effect, but was better tolerated. He was once again given rivastigmine, but experienced side effects. The patient then took galantamine and it showed a beneficial therapeutic effect for 18 months. Finally, the patient received rivastigmine and then galantamine, which resulted in partial remission of MDD symptoms. The researchers suggest that these inhibitors may act beneficially in the treatment of pattern hallucinations that occur in MDD.

Moreover, donepezil was attempted to be used in the treatment of treatment-resistant BPAD. For this purpose, eleven patients were administered the drug for 4.5 weeks [355]. The patients (*n* = 6) showed significant improvement at the donepezil 5 mg dose. In three patients, no improvement was obtained even at the higher dose of the drug. Two patients experienced side effects. In this study, it is unknown whether the improvement observed was not consistent with partial remission of patients. In another study, donepezil as an add-on treatment for mania had no beneficial effect on the therapy of treatment-resistant mania [356]. A high dropout rate was also reported. Donepezil was also administered for 4 weeks to support lithium treatment of acute mania [357]. Patients initially received a dose of 5 mg, but this dose was then increased to 10 mg. Donepezil treatment did not provide any additional benefit to patients during acute mania during prolonged use. The researchers noted improvement only after the first day of use. In one case report, donepezil induced mania in a 68-year-old female patient who had suffered a cerebellar stroke 8 years earlier [358]. AD was suspected in the patient. However, the mania did not resolve after donepezil was stopped in this patient. Similar findings were obtained in other case reports [359]. Donepezil also induced mania symptoms in patients with dementia. In a patient with AD who had a history of MDD, donepezil also induced mania [360]. The patient had no history of BPAD. Donepezil’s ability to exacerbate mania was also confirmed in a vascular dementia patient with BPAD [361]. This is also supported by another case report in which a 76-year-old patient with small-vessel cerebrovascular disease experienced BPAD type II after donepezil treatment [362]. However, she had previously experienced an episode of depression following the death of her husband. Stopping donepezil treatment caused the patient to experience a depressive-like disorder. In addition, donepezil increased BPAD type I symptoms—more specifically, depressive symptoms [363]. However, all side effects resolved after donepezil was stopped. In BPAD type II and Not Otherwise Specified (NOS) type, it was effective in treating cognitive impairment. It demonstrated a risk of destabilization of 2% and a low side effect profile requiring discontinuation of treatment of 14%. By contrast, in another study, donepezil administered for 12 weeks caused no beneficial or negative effects on late-life cognitive impairment in BPAD [364]. However, seven of nine patients reported that they noticed beneficial effects after taking the drug, although the researchers are unsure whether this was related to a placebo effect.

In one case report of a patient with AD with no previous history of mood disorders, the patient developed a manic episode after 3 days of rivastigmine treatment [365]. The patient was then prescribed donepezil and did not develop symptoms of mania again after 1 month of its administration. Another study administered rivastigmine as adjunctive treatment for BPAD during acute mania [366]. Patients also received sodium valproate. Patients were taking rivastigmine for 24 days at a dose that was 1.5 mg at first and then increased to 3 mg. Rivastigmine improved mania symptoms in these patients compared with placebo, but this improvement was not significant. In addition, rivastigmine proved effective in a patient who developed initial cerebral atrophy and treatment-resistant BPAD [367]. This drug was used in combination with oxcarbazepine and clozapine. The patient’s mood stabilized, delusions and psychomotor agitation disappeared and her sleep-wake cycle improved after taking this combination of medications. There was also a decrease in the patient’s inflammation, which was present before starting therapy. Other case reports have administered galantamine and rivastigmine and demonstrated that AChE inhibitors could exacerbate mania in BPAD type I patients with AD [368].

Galantamine was also administered as an adjunctive treatment in BPAD to improve cognitive function in these patients [369]. There was improvement in episodic memory, but not in processing speed. The patients remained on the psychotropic medications they were taking. Another study confirmed positive effects of galantamine on cognitive abilities in patients with BPAD [370]. In this study, patients also remained on their current treatment. Galantamine also had positive effects on neuronal viability and lipid membrane metabolism in the left hippocampus. In another study, galantamine also had beneficial effects on improving cognitive function as an adjunctive treatment for BPAD. However, galantamine was not effective in improving mood in these patients [371]. In one case report of a patient with a history of BPAD, galantamine as an adjunctive treatment improved mood, processing speed and attention span about 4–5 weeks after starting the treatment [372]. In addition, galantamine was well tolerated by the patient. The improvement of these functions could be responsible for the real antidepressant effect of galantamine, or the improvement of cognitive abilities could be related to the cessation of the depressive episode, or the patient also suffered from AD (galantamine is also effective treatment in AD).

In addition, fluoxetine, sertraline and amitriptyline, the standard drugs used in antidepressant therapy, inhibit blood cell AChE activity [373].

## 5. Multi-Target Directed Ligands

**Figure 3:**
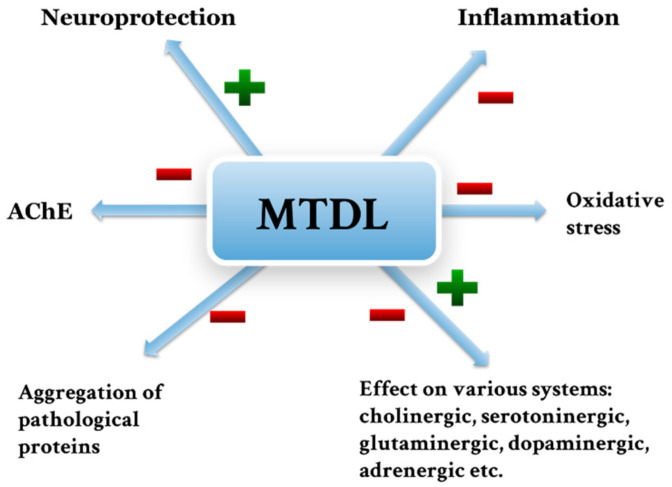
Strategies for MTDLs. “−”—inhibitory effect; “+”—beneficial effects.

Neurodegenerative diseases have a complex pathogenesis, but their progression also depends on many factors. Few effective drugs are currently available on the market that actually target the cause rather than acting only symptomatically. Therefore, new possibilities in the treatment of these diseases are being sought. This is why the term Multi-Target Directed Ligand (MTDL) was coined [374,375]. These are such compounds that target several therapeutic targets that are responsible for a particular disease entity. MTDLs are compounds that have simultaneous anti-inflammatory, antioxidant and anticholinesterase effects. They are also compounds that affect other systems such as the serotonergic, dopaminergic and glutaminergic systems (Figure 3).

An important role in the design of MTDLs is played by in silico modeling, but also by targeted experimental studies [374,375]. They allow the selection of appropriate strategies and therapeutic targets. They also clarify the mechanisms occurring in particular disease entities. MTDLs can be divided into codrugs and hybrids. Codrugs are covalent combinations of two drugs acting synergistically, mainly to improve the delivery of one or both drugs. On the other hand, hybrids, unlike codrugs are linked permanently and do not undergo enzymatic cleavage. They act on two biological targets.

Finding good MTDLs targeting several therapeutic targets is very difficult. Many constraints accompany the design of such compounds [376]. It is important that the structural fragments of MTDLs are compatible with each other, but many designed MTDLs do not have drug-like physicochemical parameters. Newly designed compounds should have good oral bioavailability and also penetrate well into the CNS. The researchers suggest that when designing MTDLs, the strategy of merging rather than fusing or linking should be used as only it will give a chance of good oral bioavailability. In addition, it should be noted that MTDL should have balanced activity on multiple targets (not always balanced in vitro IC50 values of two compounds will induce equivalent therapeutic effects in vivo). It is also important that MTDL is administered at the right time of the disease (depending on the stage of the disease, e.g., compounds that have βA as one of their targets should be administered in the prodromal stage of AD, and compounds targeting transmission in later stage of the disease). Furthermore, MTDL should not combine compounds with and without receptor-mediated mechanism of action because this will not result in balanced therapeutic effects (this is due to the different dosing to achieve the respective targets of the two classes of compounds). In addition, if an MTDL contains both receptor-mediated compounds in its molecule, attention should be paid to the nature of binding of the MTDL components to the receptor, i.e., reversibly or irreversibly (different pharmacokinetic and pharmacodynamic parameters). The combination of two compounds with different natures of binding to the receptor may result in unbalanced therapeutic effect.

Only a few examples of MTDLs are discussed in this chapter. MTDLs are mainly composed of tacrine or donepezil derivatives [377].

As for donepezil (2-[(1-benzyl-4-piperidyl)methyl] -5,6-dimethoxy-2,3-dihydroinden-1-one), it is a piperidine derivative [378]. For example, one study focused on the quinazoline/quinazolinone ring and the benzylpyridinium structure [379]. The compounds were divided into two groups: those having methoxy group substituted on oxoquinazoline ring and those without methoxy group on oxoquinazoline ring. In the first group, the compound with methoxy group substituted on oxoquinazoline ring with bromine in C3 position had the best activity towards AChE. Already, the change in the bromine substitution site itself reduced the activity of the compound. In contrast, the compounds from the first group showed similar mode of action against BChE. In the second group, the compound best inhibiting AChE was the one containing chlorine in the C4 position, but it did not detect activity against BChE. In another study, dimethyl-4-(4-((5-(4-benzylpiperidin-1-yl)pentyl)oxy)phenyl)-2,6-dimethyl-1, 4-dihydropyridine-3,5-dicarboxylate was chosen as the compound most present in AD therapy [380]. It has shown multidirectional effects: anticholinesterase, antioxidant and neuroprotective, as well as inhibiting calcium flux. The study also investigated donecopride analogues in which the cyclohexyl group was replaced by m-tolyl group, making the compound active towards AChE and 5-HT6R [381]. The substitution of the benzyl group into the piperidine ring increased the ability of the compound to interact with the active center of AChE and 5HT6R, through stronger hydrophobic bonds.

Tacrine (1,2,3,4-tetrahydroacridin-9-amine) is an AChE inhibitor. It was used as one of the drugs in AD, but it was withdrawn due to its strong hepatotoxic effects [382].

Currently, attempts are being made to use its analogs in AD therapy so as to minimize side effects while maintaining the drug’s effectiveness [383]. Major modifications made to tacrine in recent years include:

- Modifications of the A ring in which it is replaced by a pyranopyrazole grouping (pyranopyrazole Tacrines);
- Introduction of a hydroxypyranone group into the molecule (pyranopyranone Tacrines);
- Introduction of a naphthalene, quinoline or naphthoquinone moiety into the molecule (pentacylcic pyranotacrines);
- Modifications of A ring, where it is replaced by nitrogen heterocycles (pyridine-, indole- and quinoxalinotacrines);
- Modifications of A ring, where it was replaced by nitrogen heterocycles and oxygen (pyrrolo-, pyrazolo-, furanotacrines and pyrazolophthalazine tacrines);
- Modification of the A ring, where it was replaced by other heterocycles (urea and thiourea tacrines);
- Addition of an amide group at the C2 position of cyclohexyl tacrine (amido-, amino- and iminotacrine);
- Modification of the aromatic ring A where it is replaced by pyranonapthalene or pyranonaphthoquinone and simultaneously modification of the aromatic center in ring B from aminopyridine to a pyrimidinone or pyrimidinimine.

Lin-Xi Wan et.al designed and synthesized 34 new N-aryltacrine derivatives, among which a compound was found that exhibited good ChE inhibition, but also significantly lower hepatotoxicity compared to tacrine (N-(4-methoxypyridin-2-yl)-tacrine). The introduction of an N-(pyridin-2-yl)- or N-(quinolin-2-yl)- substituent at the nitrogen in the C9 position was shown to improve AChE inhibition [384]. Furthermore, all N-(pyridin-2-yl)-tacrines exhibited neuroprotective properties. Hybrids of tacrine and 2-phenylbenzothiazoles were also tested [385]. All hybrids showed nearly perfect inhibitory activity against AChE. Compounds with chlorine in the tacrine residue showed better AChE inhibition than compounds without chlorine. The compound with ethyl chain linker with substitution of chlorine atom in C6 position of tacrine residue showed the best inhibitory activity. Moreover, these compounds were not eligible for oral administration. Hybrids of tacrine and benzofuran derivative also showed good AChE inhibition ability and prevented βA aggregation [386]. This combination inhibited the enzyme at its active center and PAS. Again, the compound with a chlorine substituent at the C6 position had better inhibitory properties against AChE. The propyl chain (as linker) also improved the anticholinesterase properties. The compounds containing the OH group generally had an anti-aggregative effect against βA.

Chalcones (trans-1,3-diaryl-2-propen-1-ones), among others, are described in AD therapy [387]. These are compounds belonging to flavonoids. In one study, one of the rings was replaced by a methoxy group or an additional tertiary amine group. The methoxy group weakened the inhibitory effect against AChE, while the ethylamine group improved the inhibitory effect of the compound significantly. In addition to the introduction of a group instead of a ring, the meta and para positions were also modified. Depending on the substitution site, the compounds showed different inhibitory potentials towards AChE. As far as BChE is concerned, the methoxy group caused the opposite effect than in relation to AChE. The researchers explain this relationship by the fact that the ethylenediamine group has a higher affinity for AChE, than the methoxy group of BChE. Furthermore, the N-benzylmethylamine group bound to the active center and the diethylamine group bound to the PAS of AChE of one of the compounds. The presence of an α,β-unsaturated carbonyl group was responsible for neuroprotective and antioxidant properties.

## 6. Plants Extracts and Essential Oils

### 6.1. Hydrangea spp.

Tea infusions of *Hydrangeae Dulcis Folium* are used in Asian countries as medicine. The plant contains isocoumarins, secoiridoids and stilbenes in its composition. It is considered to have antibacterial, antidiabetic and anti-allergic effects. Jayeong Hwang et.al examined the anticholinesterase activity of two major compounds derived from Hydrangea, i.e., thunberginol C (TC) and hydrangenol 8-O-glucoside pentaacetate (HGP). Both these compounds selectively inhibited AChE and BChE [388]. TC inhibited the enzymes more effectively than HGP. Molecular docking study was also performed in this research. These compounds were shown to interact non-competitively with PAS. Inflammation is an integral component of neurodegenerative disorders. It also plays a role in MDD and BPAD. Notably, Thunberginols A, B, and F have anti-inflammatory properties [389]. It has been shown that they inhibit leukocyte degranulation by releasing IL-4 and TNF-α. It is possible that TC also has such properties, but it requires more research in this direction. In addition to the mentioned effect on cytokine release, perhaps the anti-inflammatory effect could be related to AChE inhibition, and thus this compound could act on the “cholinergic anti-inflammatory pathway”.

In addition, Hydrangenol was isolated from *Hydrangea macrophylla*. It was tested for its anti-inflammatory properties [390]. It was shown to reduce nitric oxide (NO) production by inducing heme oxygenase-1 in LPS-treated microglia cells. In addition, it reduced reactive oxygen species (ROS) production, decreased intracellular calcium concentration and increased mitochondrial membrane potential in PCl2 cells [391]. The researchers suggest that hydrangenol may affect apoptosis inhibition through down-regulation of caspase 3 and Bax, as well as up-regulation of Bcl-2.

Another compound isolated from the genus *Hydrangea* with anti-inflammatory properties is Skimmin [362]. It has an inhibitory effect on the production of IL-6 and IL-1β. *Hydrangea macrophylla* extract was also tested for antioxidant properties. It was shown that it attenuated the release of ROS in liver cells [392]. In addition, there was a decrease in the gene expression and protein levels of MAPK and it blocked the expression of the post-apoptotic transcription factor caspase-3. In this study, the hepatoprotective effect and the role of the extract of this plant in modulating the MAPK/caspase-3 pathway were demonstrated. The plant contains phenols and flavonoids, which may have caused this effect. *Hydrangea paniculata* extract was also examined and demostrated cytoprotective (renoprotective), antioxidant and anti-inflammatory activities [393]. The extract of this plant contained coumarin glycosides in high amounts; mainly skimmin, apiosylmin and others.

In the future, studies on the extracts from plants of the genus *Hydrangea* would be worth extending in the direction of neuroprotective effects. Flavonoids have proven neuroprotective effects and also exert effects on reducing oxidative stress and inflammation [394]. It is postulated that they may be effective in the treatment of neurodegenerative disorders due to these properties. Moreover, in neurodegenerative disorders, there is an increased apoptosis of neurons. Therefore, it is possible that extracts from plants of the genus *Hydrangea* could act beneficially in this direction.

### 6.2. Salvia spp.

Sages are some of the most important medicinal plants grown in the world. They have been known for their health-promoting properties for centuries. They are also used as a spice in cooking. They have several health properties such as antioxidant, antimicrobial, antimutagenic, antinociceptive and anti-inflammatory effects [395,396]. They are also known for their effects against dementia and alleviating cognitive disorders.

From *Salvia officinalis*, borneol, camphor, caryophyllene, cineole, elemene, humulene, ledene, pinene and thujone as well as flavonoids (rosmarinic acid and luteolin-7-glucoside, chlorogenic acid, ellagic acid, epicatecin, epigallocatechin gallate, quercetin, rutin) and phenolic acids (caffeic acid and 3-caffeoylquinic acid), among others, were extracted [395]. It was proven that the aqueous extract of *Salvia officinalis* inhibited AChE, thus improving memory in mice [397]. It also demonstrated antioxidant properties, and the mice tested in the study had no side effects. The extract was also effective in patients with mild to moderate AD [398]. They showed improvement in cognitive abilities after 4 months of treatment. Ethanolic extracts from dried leaves of *Salvia officinalis* were studied and shown to be able to modulate mood in healthy patients [399]. The extracts showed a dose-dependent response for anti-cholinesterase activity. After the extracts were administered, there was an improvement in mood, a reduction in anxiety and an increase in satisfaction. Of importance is that *Salvia officinalis* extract acts on primarily inhibitory 5-HT1A receptors and not on 5-HT2B or 5-HT2C receptors. In contrast, the extract showed negligible effects on AChE inhibition [400]. In addition, the extract affects α2A-adrenergic, M3 muscarinic and μ-opioid receptors.

Extracts of *Salvia rosmarinus* mainly contain rosmarinic acid, carnosic acid, carnosol, caffeic acid, betulinic acid and ursolic acid [401]. The chemical profile of rosemary oil depends on various climatic, soil or latitude and longitude factors. This is quite important, since depending on where the plant was harvested for the studies, different percentages of enzyme inhibition were shown. In one study, two populations of *Salvia rosmarinus* were collected from the Ionian and Tyrrhenian coasts. The most representative compounds were 1,8-cyneol, α-pinene, camphor and trans-caryophyllene. The sample taken on the Tyrrhenian coast showed stronger AChE inhibition than the sample collected from the Ionian coast. This study also demonstrated the antioxidant properties of rosemary oil. Rosemary oil collected in Denmark demonstrated moderate AChE inhibition, i.e., 15% at a dose of 0.1 mg/mL [402]. In another study, the following compounds were identified as being responsible for the anticholinesterase properties of an essential oil (*Salvia rosmarinus* from Spain): 3-carene and 1,8-cineole [403]. The antioxidant properties were due to bornyl acetate, camphor, terpinen-4-ol and 1,8-cineole. It was also found that carnosic acid in cell cultures inhibited the secretion of Aβ1-42 [404]. In addition, the compounds (caffeoyl derivative and phenolic diterpenoids) in the essential oil of this species also inhibited p-tau aggregation and prevented the formation of β-sheets [405]. However, rosmarinic acid was the most effective (84%, while phenolic diterpenoids were 50%). The researchers suggest that it would be appropriate to look for a substance that, like rosmarinic acid, would have a catechol grouping.

The major components of *Salvia syriaca* oil are spathulenol (mainly), isospathulenol and bornyl acetate [396]. Both the extract and the oil also have a number of flavonoids and phenolic compounds in their composition. The essential oil of this plant has been shown to have a greater free radical scavenging capacity than the extract. Researchers suggest that this is due to the higher spathulenol content. However, the essential oil was characterized by low AChE inhibition potential. On the other hand, the methanolic extract had a moderate AChE inhibition potential, which was attributed to the higher content of phenolic compounds (mainly rutin, rosmarinic acid, ferulic acid, apigenin and quercetin) compared to the essential oil.

*Salvia lavandulaefolia* essential oil was tested in patients with mild to moderate AD. The oil was very well tolerated in these patients, with almost no side effects [406]. The 6-week treatment resulted in a 14.4% decrease in AChE activity. There was an improvement in patients’ scores on the Neuropsychiatric Inventory (NPI) and the Cognitive Drug Research (CDR) scales. In another study, the effects of an oil from this plant on cognitive performance and mood during different occasions 7 days apart were tested on a larger group (mean age 23.8 years) [407]. The oil, as before, proved to be a potent selective inhibitor of AChE. Significant differences were observed in improved memory, attention, reduced reaction time, improved word and picture recognition task, but also improved general cognitive ability. Patients receiving the oil rated themselves as less mentally fatigued, and improvements in mood were also noted. Importantly the oil was mainly composed of monoterpenoids such as camphor (37%), 1,8-cineole (36.4%), camphene, a-pinene, b-pinene, limonene and endo-borneol.

*Salvia fruticosa* oil was also tested on SH-SY5Y cells [408]. It was observed that the oil had neuroprotective effects by inhibiting GSK3β CK-1δ and β-secretase and increasing of p-GSK-3β protein levels. The oil demonstrated significant protection against cytotoxicity induced by Aβ 1-42. The major phenolic compounds were rosemary acid, luteolin 7-O-glucuronide and caffeic acid. In another study, *Salvia fruticosa* extracts with different polarity (solvents: methanol, dichloromethane, naphthyl ether) were tested [409]. Each extract exhibited antioxidant properties, but the methanolic extract presented the greatest ability to scavenge ROS. The experiment was conducted on SH-SY5Y cell cultures. However, the naphthyl ether extract showed the greatest ability towards protective effect against βA toxicity. No neuroprotective effect was observed for the methanol extract. The efficacy of extracts and essential oil of this sage species (wild-grown species and cultivated species) was compared [410]. The oil showed the strongest anticholinesterase activity. Wild-grown species demonstrated lower AChE inhibitory potential than cultivated species. The essential oil contained mainly 1,8-cineol, camphor and thujone in its composition. This study also reported the antioxidant properties of extracts and essential oil of *Salvia fruticosa.*

Methanolic extracts and essential oil of *Salvia chionanth* were also studied for their inhibitory effects against AChE and BChE [411]. It was shown that the extract inhibited only BChE, while the essential oil inhibited moderately both AChE and BChE (compared with the inhibition of AChE and BChE by galantamine). The essential oil also had moderate antioxidant activity.

In addition, the essential oil of *Salvia urmiensis* was also screened for use in AD. In flowers, mainly 6,10,14-trimethyl-2-pentadecanone (55.7%), 1,8-cineol (6.5%) and β-pinene (6.4%) were identified, while in leaves, ethyl linoleate (19%), methyl hexadecanoate (17%) and 6,10,14-trimethyl-2-pentadecanone (13%) [412]. The flower essential oil had better potential anticholinesterase activity than the leaf essential oil. The researchers considered monoterpenoids as the inhibitory substances contained in the oil of the enzyme because they are well known as AChE inhibitory compounds. In addition, they are found in higher amounts in the essential oil from the flowers than in the leaves.

Other sage species such as *Salvia chrysophylla* were also evaluated and proved to have anticholinesterase activity, as well [413]. The substance mainly responsible for inhibition of AChE and BChE was sclareol, while triterpenes (oleanolic and ursolic acids) had only inhibitory effects on AChE. The antioxidant activity of the extracts of this species was also demonstrated.

Cell culture studies showed that Salvianolic Acid B isolated from *Salvia miltiorrhiza* decreased extracellular βA, soluble APPβ and intracellular C-terminal fragment β from APP in a dose-dependent manner [414]. However, it did not affect α-secretase and γ-secretase activities and full-length APP levels. It was shown to interact with β-secretase 1 (BACE1) through its inhibition at the active center. It was also shown to have beneficial effects on MDD in mice exposed to FST, SPT and TST [415]. There was reduced anhedonia in the animals and less weight loss. This correlated with inhibition of M1 microglia activation. After administration of this compound, there were changes in the M1/M2 microglia ratio, contributing to increased neurogenesis. In another study, Salvianolic Acid B in a rat model of chronic stress-induced MDD showed an alleviating effect on its symptoms [416]. This was associated, as before, with a reduction in inflammation and oxidative stress. The compound also activated the adenosine monophosphate-activated protein kinase/sirtuin 1 (AMPK/SIRT1) signaling pathway. The AMPK/SIRT1 pathway also plays a role in AD [417], PD [418], SM [419], HD [420] and ALS [421]. It is possible that in the progression of these disease entities, this compound will also act on this pathway, contributing to the improvement of the patient’s condition. In addition, Salvianolic Acid B in a mouse model of AD had beneficial effects on improving memory, reducing the number of activated microglia and astrocytes, and showed antioxidant and neuroprotective effects [422]. Moreover, it reduced cognitive impairment induced by scopolamine or Aβ25-35 peptide in mice [423]. Salvianolic Acid B inhibited the formation of α-synuclein fibrils. In addition, it had a cytoprotective effect on cells by protecting against the toxic effects of α-synuclein aggregates [424]. This compound is non-toxic and had no effect on cell viability and demonstrated a concentration-dependent effect. *Salvia miltiorrhiza* also contains tanshinone I and tanshinone IIA in its essential oil composition [425]. These compounds inhibited a-synuclein aggregation and depolymerization of preformed α-synuclein fibrils in a transgenic PD model of Caenorhabditis elegans. In addition, there was an increase in the lifetime of this nematode. Tanshinone IIA acted beneficially in a rat model of AD, showing neuroprotective effects, and it improved learning ability and memory in rats [426]. Furthermore, it inhibited the expression at the transcriptional and translational levels of inducible nitric oxide synthase (iNOS), matrix metalloproteinase-2 (MMP-2) and nuclear transcription factor κ (NF-κBp65) genes. Improvements in memory, reduction in AChE activity, mitigation of neuronal damage and oxidative stress, and in restoring cholinergic balance have also been observed in other studies following administration of this compound [427,428].

The effect of *Salvia lachnostachys* leaf extract and the effect of fruticuline A contained therein on depressive behaviors in MDD and neuropathic pain was studied by experimenting on Wistar rats [429]. MDD was induced with clonidine and the neuropathic pain model was induced with formalin. It was proved in this study the antidepressant effect of the extract as well as the fruticuline A contained in it.

It is worth noting the caffeic acid found in various species of sage [430]. In PCl2 cell cultures, it showed a beneficial effect in reducing p-tau phosphorylation and attenuating the influx of calcium ions into the cell. This compound protected the cells from toxicity induced by βA.

A combination of two types of sage (Cognivia™), *Salvia officinalis and Salvia lavandulaefolia*, was tested [431]. Patients were evaluated after 120 min, 240 min, and after 29 days of taking this extract. Consistent improvements in cognitive ability and working memory were observed in healthy patients. However, no improvement was observed in mood. This is consistent with a study in which the same combination of salvia species was tested in mice exposed to Morris Water Maze Learning and the Y-maze test [432]. Chronic administration improved cognitive ability in mice, but had no effect on oxidative stress, hippocampal neurogenesis or neuronal activity. The animals exhibited increased calmodulin-dependent protein kinase II (CaMKII) expression, but no increase in BDNF expression. In addition, improvements in spatial memory were also noted in chronic and acute administration.

A combination of *Salvia officinalis*, *Salvia Rosmarinus* and *Melissa officinalis* was also evaluated for cognitive improvement in healthy patients taking an ethanolic extract of these plants for 2 weeks [433]. The combination was effective in improving verbal episodic memory. Significantly, no side effects were noted.

As for ALS, sage has also been tried in the treatment of this condition. KCHO-1 is an ethanol extract of nine herbs: *Curcuma longa*, *Salvia miltiorrhiza*, *Gastrodia elata*, *Chaenomeles sinensis*, *Polygala tenuifolia*, *Paeonia japonica*, *Glycyrrhiza uralensis*, *Atractylodes japonica* and processed *Aconitum carmichaeli* [434]. The mixture of these herbs reduced oxidative stress and prevented neuronal degeneration in a mouse model of ALS. Its administration delayed disease progression and also improved motor activity in mice. The combination of herbs reduces oxidative stress by decreasing the expression of gp91 phox and affecting the MAPK pathway, and improves the survival rate. In addition, the mixture of these herbs reduces microglia activation and proliferation. There are also registered clinical trials in patients with ALS who are taking a mixture of these herbs and the standard treatment received for ALS [435].

A combination of *Salvia miltiorrhiza* and *Panax ginseng* was studied in a mouse model of R6/2 HD. It demonstrated cardioprotective effects in these animals by reducing apoptosis and oxidative stress and inflammation [436]. The cardioprotective effect was also associated with inhibition of HTT aggregation.

There are also ongoing clinical trials of salvia formulations and their therapeutic uses. MDD is in phase III clinical trials, while AD, PD and BPAD are in phase II clinical trials [437].

### 6.3. Prunus spp.

Various species of *Prunus* spp. have also been studied for use in AD. In one study, the essential oils of *Prunus armeniaca* and *Prunus domestica* were analyzed [438]. The fruit of *Prunus armeniaca* contained mainly phytol, heptacosane, nonacosane and other compounds, while the leaves of the second species contained mainly pentacosane, phytol, nonacosane and others. Depending on the harvesting time, the essential oils had different percentage composition as to the percentage content of each compound. Concentration-dependent antioxidant capacities were observed for both oils. *Prunus domestcia* oils had higher free radical scavenging capacity than *Prunus armeniaca*. The researchers suggest that the antioxidant abilities are responsible for the phytol contained in both oils. In addition, the oils showed neuroprotective effects. It is worth noting that *Prunus armeniaca* oils inhibited AChE more effectively, while *Prunus domestcia* inhibited BChE. The enzyme inhibition ability also differed according to the harvesting time of leaves or fruits. Furthermore, extracts from freshly collected *Prunus domestcia* fruits exhibited a greater ability to scavenge free radicals than extracts from dried fruits [439]. Its extracts have also been shown to have anti-inflammatory effects and may reduce conditions associated with pain [440]. Chronic pain is a contributing factor to depressive disorders. The properties of this extract would be worth studying for use in the treatment of MDD [441]. Of note, *Prunus domestica* fruits contain chlorogenic acid. It was demonstrated that this compound had both antioxidant and anti-anxiety effects in mice. In addition, aqueous-alcohol extracts from this plant had beneficial effects on memory and learning in rats after 7 days of administration [442].

Extracts from bitter and sweet apricot kernels of *Prunus armeniaca* were also studied for AChE inhibition [443]. The inhibitory activity against cholinesterases was evaluated in comparison with rivastigmine. The aqueous ester of bitter apricot kernel had the best AChE inhibitory potential, while the extract with moderate activity on AChE was aqueous extract of sweet apricot kernel. Other extracts (ethanolic, water–ethanol, naphthyl) examined in this study exhibited low or no inhibitory activity against AChE. The effect of amigdalin on AChE was also studied, however, it showed no anticholinesterase activity. None of the extracts had any effect on BChE in this study. Both aqueous extracts showed neuroprotective effects on PCl2 cells. However, the sweet kernel extract showed a better neuroprotective effect. Furthermore, it was shown that the amount of phenols and flavonoids contained in both aqueous extracts was independent of the activity against AChE. As mentioned earlier, *Prunus armeniaca* extracts have anti-inflammatory, antioxidant and antinociceptive effects [444,445,446].

*Prunus amygdalus* was also studied for its use in AD [447]. The effects of nuts on cognitive function and on AChE activity were studied in rats in which amnesia was induced with scopolamine. Nut paste was administered orally for 7 and 14 consecutive days. The administration of the paste was not accompanied by any side effects. The rats showed improved memory, but also reversal of scopolamine-induced amnesia after administration of the paste. It also exhibited anticholinesterase properties, since during its administration, there was a decrease in AChE activity. In addition, the composition of almond hulls contains phenols, which makes them antioxidant [448].

*Prunus mume* has also been studied in the context of AD [449]. For this purpose, ethanolic extracts of the fruits of this plant were administered to mice in which amnesia was induced with scopolamine. Mice receiving the extract showed improved cognitive ability and increased ChAT expression. The extract attenuated the scopolamine-induced increase in AChE activity. In another study on 5XFAD transgenic mice, similar effects were also observed [450]. Its fruit was shown to enhance the cholinergic action of neurons. In addition, extracts from the fruits of this plant were also studied in rats with induced cerebral hyperfusion [451]. The extract alleviated microglia activation, benefited cognitive deficits, affected MAPK signaling and attenuated NF-κB activation (regulates apoptosis). Besides inhibiting microglia activation, the extract also attenuated the expression of pro-inflammatory cytokines such as IL-1β and IL-6 and cyclooxygenase-2 [452]. It also reduced ChAT expression in the medial septum and hippocampus. In addition, extracts of other *Prunus* spp. such as *Prunus spinos* showed antioxidant and anti-inflammatory properties [453,454]. Zhengtian Capsule is a Chinese patent medicine that consists of a mixture of 15 herbs including *Prunus persica* [455]. It has been shown that this drug can alleviate symptoms similar to oxidative stress depression, increase proliferation of neural stem cells and neurons, promote levels of BDNF, phosphorylated ERK1/2 and CREB and suppress expression of NF-κB.

Prunus armeniaca is part of the Kampo Zokumei-to formula [456]. The mixture includes eight other herbs, such as Ephedra sinica, Cinnamomum cassia, Panax ginseng, Angelica acutiloba, Cnidium officinale, Zingiber officinale and Glycyrrhiza uralensis. This mixture was shown to improve memory after Aβ25-32 injection in mice. In addition, there was a restoration of adequate SYN levels in the cerebral cortex and hippocampus.

### 6.4. Citrus spp.

The use of *Citrus* spp. has also been attempted in AD therapy. Plants from this species are rich in hesperdin, naringin, narirutin, neohesperidin and limonoids. In the context of AD treatment, the effects of *Citrus aurantium* seeds were tested on rats in which AD was induced with AlCl3 [457]. Compounds in the seeds including hesperdin and limanoids had a protective effect on the cognitive abilities that were impaired by AlCl3 administration. An improvement in OFT was also observed. The effects of high-dose limanoids and hesperdin (given in two doses) showed effects almost identical (or even better) to donepezil. The researchers suggest that hesperdin disrupts the deposition of βA in the brain through an immune mechanism (reducing the amount of factor β1). The limanoids in the seeds had a probable neuroprotective effect. These compounds also affected AChE and βA levels in the brain. The anticholinesterase and antioxidant abilities of essential oils from the peels of three types of citrus were also studied: *Citrus aurantifolia*, *Citrus aurantium* and *Citrus bergamia* [458]. The oils mainly contained limonene, α-pinene, β-pinene, γ-terpinene and linalyl acetate. The essential oils showed a concentration–response relationship in their antioxidant properties. *Citrus aurantifolia* oil had the best free radical scavenging activity. Limonene contained in the oil is most responsible for the antioxidant capacity. A concentration–response relationship was also observed for AChE inhibitory activity. The best activity against AChE was demonstrated by the oils from *Citrus aurantifolia* and *Citrus aurantium*. The monoterpenes contained in the oils were responsible for the anticholinesterase activity. In another study, various extracts of *Citrus aurantifolia* leaves and peels collected in different geographical regions of Italy were studied [459]. The extracts showed phytochemical variability. The most abundant compounds were apigenin, rutin, quercetin, kaempferol and nobiletin. Methanolic extracts had strong antioxidant properties and one of them had the highest selectivity in inhibiting AChE. In contrast, the n-hexane extract showed the highest inhibitory potential against both AChE and BChE.

A methanolic extract from *Citrus junos* was also studied for AChE inhibition. It showed a significant effect on AChE inhibition in vitro [460,461]. The compound responsible for this property was naringenin. It inhibited the enzyme in a dose-dependent manner. Naringenin administered to mice with induced amnesia (using scopolamine) resulted in relief of its symptoms. The compound also has good antioxidant properties, i.e., reduces ROS and increases antioxidant activity [462]. In addition, in a rat model of AD, the compound showed improvements in learning and memory abilities, as well as antioxidant properties [463]. Naringenin also reduced the process of apoptosis in the hippocampus. The researchers suggest that the interaction of the compound with the estrogen receptor (antagonistic effect) was responsible for some of the beneficial effects. In another study, pretreatment of animals with this compound before inducing neurotoxicity with AlCl3/D-galactose caused fewer behavioral changes in the animals as well as less memory impairment than in the group not receiving this compound [464]. The compound also affected the cholinergic system through higher levels of ACh and lower levels of AChE relative to the group not previously receiving naringenin. In other studies, it also showed positive effects on memory and learning in rats, antioxidant properties and also neuroprotective properties [465,466,467]. Furthermore, Naringenin ameliorated the neurotoxic effects of βA and reduced amyloidogenesis (decrease in APP and β-secretase expression) [468]. It is noteworthy that in addition to these actions, it also affected the levels of p-tau. In addition, the compound inhibited βA1-42-induced M1 microglia activation and promoted M2 microglia polarization [469]. The researchers demonstrated that this is one of the few compounds that increases the expression of βA degrading enzymes such as neprilysin and insulin degradation enzyme. This compound also acted beneficially on memory impairment in type 2 diabetes through AChE inhibition and antioxidant activity [470].

The properties of naringenin were also tried in induced PD. The compound acted as a cytoprotective effect, protecting cells from the effects of 6-hydroxydopamine [471]. In cell culture, it activated the Nrf2/ARE pathway and affected Nrf2/ARE protein levels in mice. The compound also caused an increase in NrF2 transcript. In another study, this compound affected body weight, locomotor ability and the expression of parkin, PARK 7 protein, tyrosine hydroxylase and C terminus Hsp70 interacting proteins in the striatum and SN in induced PD in animals [472]. It showed neuroprotective and anti-apoptotic effects. In addition, it reduced α-synuclein expression and had anti-inflammatory effects in a mouse model of PD, as well as benefiting dopamine turnover [473]. Cell cultures and Zebrafish model also showed beneficial effects in 6-hydroxydopamine-induced PD [474]. The compound affected the swimming pattern in Zebrafish larvae and caused altered expression of casp9, lrrk2 and polg (down-regulation) and pink1 (up-regulation). In addition, it affected oxidative stress and showed cytoprotective effects. Moreover, it demonstrated anti-inflammatory and protective effects on dopaminergic neurons against LPS-induced toxicity [475]. It also inhibited the activation of microglia and the NLRP3 inflammasome. The neuroprotective and antioxidant effects in PD of naringenin were also proved by another study [476]. PD was induced in *Drosophila melanogaster* and in Wistar rats. The researchers also observed that the compound is a molecule that passes potentially through the blood–brain barrier. In rodents, locomotor ability was improved, and in insects, the disease-inducing effect was reversed. Notably, this compound exhibited a protective effect on neurons against 3-NPA-induced toxicity [477]. Naringenin also increased serotonin levels in the striatum and increased monoamine oxidase activity. In animals, there was an attenuation of behavioral changes.

Due to its anti-inflammatory properties, this compound has also been tested in a mouse model of SM [478]. The compound was shown to delay the onset of the disease, reduce the incidence of the disease and alleviate its symptoms. It also acted as an anti-inflammatory by affecting the levels of pro-inflammatory CD4+ T cell subsets Th1, Th9 and Th17 cells together with their respective transcription factors T-bet, PU.1 and RORγt. In addition, it decreased demyelination, reduced inflammatory infiltration in the spinal cord and decreased plasma levels of cytokines such as TNF-α and IL-6. In addition to the aforementioned properties, this compound also showed protective effects on the blood–brain barrier in model animals [479]. It also affected disease progression and reduced levels of the cytokines IFN-γ, IL-17 and IL-6, as well as blocking chemotaxis and antigen presentation. Naringin and naringenin have also been tested for use in ALS [480]. These compounds have been shown to affect superoxide dismutase Cu/Zn-1 (SOD1). Mutations in the gene of this protein are thought to be one of the causes of ALS. Naringin was better at binding to and inhibiting the formation of toxic SOD1 aggregates than naringenin. Naringenin also showed beneficial effects in ameliorating depressive and anxiety-like behaviors in hypoxic stressed animals [481]. It acted on oxidative stress, pro-inflammatory cytokine production and NF-kB/BDNF expression. In addition to its anti-inflammatory effect, its antinociceptive activity has been demonstrated [482]. It has been repeatedly emphasized that pain is accompanied by depressive disorders. The beneficial effect on depressive symptoms may be due to this property [483]. It has also been demonstrated that this compound influences serotonergic and noradrenergic transmission, thus reducing MDD symptoms. Animals were evaluated during TST, FST and OFT. However, the compound had no effect in the FST and OFT, while statistical significance was found in the TST. In another study in olfactory bulbectomized-mice, which are animal models of MDD, naringenin showed a beneficial effect in reducing depressive symptoms [484]. However, in this study, the beneficial effect was observed in all tests: SPT, FST and OFT. The compound also had anti-inflammatory effects, reducing proinflammatory cytokines and positively reducing oxidative stress, as well as significantly reducing NF-kB levels. The antidepressant effect was also confirmed in another study in TST in mice [485]. The compound affected the glucocorticoid receptor in the hippocampus, as well as monoamine neurotransmitter levels and decreased serum corticosterone levels.

Completely different results in the context of AChE inhibition were obtained after testing 17 extracts from different *Citrus* spp. Extracts in concentration of 500 µg/mL had no inhibitory effect on AChE [486]. Some of the extracts inhibited BChE. Interestingly, hesperidin at 100 µg/mL showed only 27.3 ± 1.2% inhibition of AChE and 17.7 ± 4.2% inhibition of BChE. The anticholinesterase properties of the flavanones hesperetin, naringenin and hesperidin were also studied [487]. It was shown that hesperidin had inhibitory effects on both AChE and BChE, while the other two compounds were more specific to AChE. In addition, these three compounds acted non-competitively to inhibit BACE1. It was shown that there was a correlation between the sugar groupings in the compound molecule and the potency of interaction with AChE. It was also shown that hesperidin alleviated non-cognitive deficits in APP/PS1 transgenic mice after only 10 days of its administration [488]. The compound also attenuated βA deposition and, APP expression in the cortical region, and decreased microglia activation and TGF-β immunoreactivity. However, APP expression levels and βA levels decreased after treatment in the hippocampus, but this was not statistically significant. Hesperidin in a mouse model of AD improved memory and learning ability [489]. There was also an improvement in locomotor ability and an increase in antioxidant defense, via inhibition of GSK-3β, and a consequent reduction in mitochondrial dysfunction. Hesperidin failed to demonstrate activity in the context of βA deposition. However, after treatment, there was a reduction in soluble and insoluble βA (decreased levels of soluble βA1-40 in the cortex and hippocampus, and decreased insoluble βA1-40 in the hippocampus). However, in the cortex, the level of insoluble βA1-40 did not decrease. Furthermore, hesperidin did not affect the levels of soluble and insoluble βA1-42 in the cortex and hippocampus. In another study of hesperidin in a rat model of AD (AD was induced with aluminum chloride (AlCl3)) [490], administration of the compound reduced memory and learning defects. In addition, treatment decreased apoptosis and oxidative stress in the cortex, cerebellum and hippocampus of the animals by decreasing Bax expression and increasing Bcl-2 expression. Furthermore, hesperidin reduced Tau and Aβ pathologies, in addition to having anti-inflammatory and anti-apoptotic effects and affecting the AKT/GSK-3β pathway exerting neuroprotective effects [491]. Mitochondrial voltage-dependent anion channel 1 (VDAC1) is involved in AD pathology. Hesperidin increased the phosphorylation level of VDAC1, indicating its anti-apoptotic effect in PCl2 cells [492]. In addition, hesperidin has been shown to act to increase the synaptogenic ability of cortical astrocytes by modulating astrocytic TGF-β1 signaling, and also acts by inducing and forming synapses in neurons of the hippocampus and cortex [493].

Moreover, this compound was tested for PD treatment. For this purpose, the disease was induced by 6-hydroxydopamine in mice [494]. It was observed that hesperidin reduced memory impairment and depressive behavior, and also attenuated the effects of 6-hydroxydopamine. In another study, in which PD-like symptoms were also induced, 6-hydroxydopamine hesperidin improved motor, olfactory and spatial memory impairments [495]. In addition, it acted beneficially by preventing the loss of dopaminergic neurons and prevented the depletion of dopamine and its metabolites. In addition, it prevented mitochondrial dysfunction by inhibiting respiratory chain complexes I, IV and V. The compound also regulated the apoptosis pathway. Hesperidin showed antidepressant and anti-anxiety effects in induced PD (with 6-hydroxydopamine) in rats [496]. The administration of this compound induced a decrease in the levels of proinflammatory cytokines such as TNF-α, INF-γ, IL-1β, IL-2 and IL-6. Hesperidin increased the expression of neutrophin-3 (NT-3), nerve growth factor (NGF) and BDNF. In addition, dopamine and its metabolite in the striatum increased after treatment. Hesperidin had a neuroprotective effect on dopaminergic neurons in SN. Hesperidin was also tested in a model of PD in Drosophila melanogaster [497]. The compound restored normal dopamine levels, normal AChE activity and improved motor function. It also improved the survival rate of the insects and had antioxidant and antioxidant effects. Hesperidin reduced levels of lrrk2 and gsk3β kinases, which are thought to be involved in α-synuclein deposition, and p-tau [498]. The beneficial effects of hesperidin in PD were also observed in other studies [499,500,501]. This effect was mainly due to the mechanisms and properties described above. The treatment of HD with hesperidin has also been attempted [502]. Mice were first treated with 3-NPA and then administered with hesperidin. This compound abolished the changes caused by 3-NPA. It showed neuroprotective and anti-inflammatory effects. Pretreatment with hesperidin prevented changes in locomotor activity or prepulse inhibition. There was also a smaller increase in malondialdehyde and a smaller decrease in catalase activity compared to mice that did not receive treatment. This study also demonstrated the neuroprotective properties of this compound. In another study, similar results were obtained [503]. It was also noted that the mechanism of action of hesperidin may be related to NO.

This compound is worth looking at for SM, as many pro-inflammatory cytokines are increased in this disease. Through its anti-inflammatory effects, hesperidin may work to benefit the treatment of SM by decreasing the inflammatory response, which may help to reduce disease progression. Hesperidin was tested in a mouse model of SM (Mice with Experimental Autoimmune Encephalomyelitis) [504]. The compound attenuated disease progression, reduced neuronal demyelination and acted to regulate levels of pro-inflammatory and anti-inflammatory cytokines. A reduced number of actively proliferating T lymphocytes was also observed, and consequently, a change in the polarity of CD4+ T lymphocytes toward regulatory T cells. Similar observations have been noted in other studies in mouse models of SM [505,506]. Treatment with this compound prevents oxidative stress damage.

Hesperidin also showed antidepressant and anti-anxiety effects in a rat model of diabetes (induced by streptozocin) [507]. The animals were submitted to the tests OFT, FST and Elevated Plus Maze. Furthermore, its neuroprotective effects were shown to be due to its action through activation of Nrf2 signaling. It has also been shown that its antidepressant effects may be due to its interaction with serotonergic 5HT1A receptors [508]. Hesperidin was administered to mice that showed reduced immobility time in the FST and TST after its administration, with no effect on motor activity in the OFT. Stress is one of the risk factors associated with neurodegenerative diseases and depressive disorders. Animals subjected to acute stress reaction (immobilization for 6 h) showed restlessness, locomotor disorders and anxiety-like behavior [509]. A 14-day pretreatment with hesperidin inhibited the mentioned symptoms to some extent. However, the drug did not completely reverse the stress-induced changes compared to naive animals. The compound had an antioxidant effect as in the previously mentioned studies. Hesperidin did not affect mitochondrial enzyme complexes I, II and IV, but acted against azotergic stress. In mice with mild TBI, hesperidin showed antidepressant effects and decreased proinflammatory cytokines such as IL-1β, TNF-α and malondialdehyde levels, and also increased BDNF levels [510]. Thus, hesperidin acted beneficially in PTSD by reducing depressive behaviors in OFT and FST in rats [511]. This study also showed that the compound may modulate serotonin signaling, by which it would affect the serotonergic system. This compound acted beneficially to treat cognitive impairment and depressive symptoms in a mouse model of MDD (induced by olfactory bulbectomy) [512,513]. It exerted beneficial effects by regulating the levels of AChE and pro-inflammatory cytokines, as well as the levels of NGF and BDNF.

Another study demonstrated the cytoprotective effects of hesperetin and hesperidin. This study exploited the fact that βA deposition may be associated with impaired neuronal energy metabolism [514]. After treating the cells, which were previously treated with Aβ1-42, with hesperetin and hesperidin, an increase in glucose uptake was observed. These compounds were shown to improve the impaired energy metabolism, which may reduce βA-induced neuronal damage. It is noteworthy that hesperetin exhibits anti-inflammatory properties, acting by reducing the levels of IL-1β and IL-6. The compound was also shown to protect neurons from βA-induced toxicity in cell culture [515]. The compound attenuated oxidative stress and reduced Aβ pathology. It also showed anti-apoptotic effects and affected microglia and astrocyte activation and TLR4 expression. Reduced expression of APP, BACE1 and Aβ and increased levels of synaptic markers were also observed. In addition, mice treated with this compound had improved cognitive abilities. Notably, this compound exhibits anti-inflammatory properties by acting by reducing IL-1β and IL-6 levels [516]. Hesperetin was also shown to inhibit microglia and astrocyte activation, NO production and inducible nitric oxide synthase expression, and to reduce ERK phosphorylation.

Furthermore, hesperetin showed beneficial effects in rats with induced PD by 6-hydroxydopamine [517]. This compound showed an anti-apoptotic effect on dopaminergic neurons and attenuated the effects of oxidative stress and astrogliosis. In addition, rats treated with hesperetin improved their motor skills. It was shown that this compound may be useful in the early stages of PD as an adjuvant. In other studies, this compound showed neuroprotective (6-hydroxydopamine-induced toxicity) and anti-apoptotic effects (decreased levels of cleaved caspase 3 and caspase 9), as well as antioxidant effects through activation of the NRF2/ARE pathway [518]. In a rat model of PD, it also showed antioxidant activity by increasing levels of catalase and SOD1 [519]. Moreover, hesperetin in combination with pyridoxine showed antioxidant activity in rats with induced PD [520]. These two compounds in combination positively regulated the levels of enzymes such as catalase, superoxide dismutase, glutathione and positively affected the levels of ACh and dopamine.

Hesperetin therapy has been reported to attenuate LPS-induced changes, including anti-inflammatory and antiapoptotic effects, decreasing ROS production, increasing levels of antioxidant proteins and cytoprotection [521]. Moreover, hesperetin improves synaptic integrity, cognition, learning ability and memory by enhancing the phosphorylated-cAMP response element binding protein (p-CREB), postsynaptic density protein-95 (PSD-95) and syntaxin. In addition, it reduced microglia and astrocyte activation by downregulating the expression of GFAP and Iba-1, thus modulating TLR4/NF-κB signaling. In addition to its beneficial anti-inflammatory effects, hesperetin has been shown to reduce neuronal demyelination, i.e., protect and repair the myelin sheath. This property may account for the efficacy in SM therapy [522]. This compound also exhibits antidepressant properties as demonstrated in rats with induced MDD [523]. They were tested by FST, OPT, SPT and Elevated Plus Maze Test. The animals showed a reduction in anxiety and apprehension, as well as an alleviation of depressive symptoms. Hesperetin derivatives were also attempted so that these compounds inhibit AChE doubly, i.e., binding to the active center and to the PAS. The derivative with the highest selectivity and AChE inhibitory potential was 2-[5-Hydroxy-2-(3-hydroxy-4-methoxyphenyl)-4-oxochroman-7-yl]-N-(4-trifluoro-methylbenzyl) acetamide [524]. This compound had an electronegative group at the para position of the benzene ring, and the electronegativity of the substituent was higher. This property probably made the compound bind to AChE at both sites. It showed neuroprotective and antioxidant activity. It was also one of the derivatives that showed the strongest inhibitory effect on spontaneous aggregation of βA.

As aforementioned, narirutin is one of the compounds isolated from *Citrus* spp. There has also been an attempt to use this compound in AD therapy. It was shown that this compound had a high potency to inhibit βA aggregation [525]. Moreover, it bound to the active site of BACE1, changing the form of the enzyme so that it could not recognize the substrate. It was also characterized by moderate antioxidant properties. In addition, the compound is characterized by low toxicity. In a mouse model of chronic mild stress, it alleviated depression-like behaviors after one week of use [526]. It also showed no side effects while possessing anxiolytic.

### 6.5. Xanthones

Xanthones are compounds that are found in higher plants, lichens and fungi. Their chemical name is dibenzo-γ-pyrone. Their derivatives are gaining popularity for their anticholinesterase properties. We can artificially divide them into compounds of natural origin and synthetic compounds [527,528].

Due to the structure of these compounds, such modifications can be referred to as MTDLs. In one study, all obtained synthetic derivatives of xanthones inhibited AChE, including one that inhibited the enzyme comparably to tacrine [529]. They also interacted with the active center and the PAS of AChE, and had higher antioxidant properties than vitamin C. The alkoxy or alkenoxy substituents in these compounds at position 3 were also shown to affect the potency of ChE inhibition [530]. As before, the derivatives bound to both the PAS and the active center. Derivatives having a dialkylamine methyl at the side chain end at position 2 in xanthones showed higher inhibitory activity. In another study, xanthones derivatives showed potent effects on AChE and on monoamine oxidase. In addition, they inhibited the induced aggregation of βA1-42 [531]. They also showed low toxicity in cell cultures, and they bound to the active site and PAS of the enzyme and could pass well through the blood–brain barrier. The strength of enzyme inhibition was related to the length of the alkylene spacer chain. The derivatives also exhibited effects on BChE, but weaker than those against AChE. Derivatives of xanthones have also been studied in many other studies [532,533,534,535,536]. The results obtained in them were similar to each other. These derivatives showed anticholinesterase, antioxidant and anti-inflammatory effects.

As for natural xanthones, several different compounds have been isolated from the fungus Amauroderma amoiensis [537]. Among them was xanthone. It had a weak inhibitory effect on AChE (<10%). Xanthones were also isolated from Centaurium erythraea leaf extract [538]. These compounds had good antioxidant activity but did not significantly scavenge the NO radical. Nevertheless, they strongly inhibited AChE. Centarium umbellatum extracts were tested in another study [539]. As before, they inhibited AChE and had antioxidant effects.

Mangiferin is a compound belonging to natural xanthones. In studies, it has exhibited cytoprotective effects [540]. In addition, the compound reversed the amnesia and learning impairment induced by scopolamine in rats, and it showed anticholinesterase and antioxidant activities [541,542]. The compound also was characterized by low toxicity. In one study, the compound did not affect βA but did affect p-tau, reducing its hyperphosphorylation in the cortex and hippocampus [543]. In addition, it had an anti-inflammatory effect by reducing activation of microglia and astrocytes, and alleviated neuronal damage in a mouse model of AD. They also experienced improvements in episodic and spatial memory. In addition, mangiferin was also shown to have beneficial effects in mice with PD [544]. It attenuated dopaminergic neurodegeneration and motor impairment. It restored redox balance and affected the expression of Bcl-2/Bax. This compound also has anti-inflammatory properties because it decreased the production of NO and pro-inflammatory cytokines such as IL-1β, IL-6 and TNF-α [545]. It also promoted the polarization of inflammation in the anti-inflammatory pathway and inhibited the activation of NF-κB. In another study, cognitive deficits were induced with quinolinic acid. This compound induced motor and cognitive impairment [546]. In rats treated with this acid, mangiferin reversed the effects induced by this acid. After 21 days of treatment, mangiferin also decreased AChE activity in the hippocampus and striatum and reduced levels of IL-1β, as well as TNF-α. Mangiferin pretreatment reduced anxiety-like behavior and LPS-induced anhedonic behavior in mice [547]. It also reduced depressive symptoms and oxidative stress. Moreover, it exerted anti-inflammatory effects by affecting IL-1β levels without significant effects on TNF-α, and also prevented declines in BDNF levels.

Furthermore, it was shown that mangiferin and morin exhibited antioxidant and antioxidant effects and protected neurons from βA-induced toxicity [548]. They acted on some forms of apoptosis (reducing caspase 3 levels), but also alleviated mitochondrial dysfunction. As for morin, it was shown to counteract neuropathological and cognitive changes in AD model mice [549]. It improved memory and spatial learning impairments, thereby reducing βA production and platelet load in these animals. The researchers suggest that this effect can be attributed to the compound’s action on the non-amyloidogenic pathway by affecting α-secretase and its action on the amyloidogenic pathway by affecting β-secretase. Furthermore, the compound ameliorates tau hyperphosphorylation, reduced microglia activation and synaptic deficits, and inhibited the expression of BACE1 and PS1. In another study, the compound reduced p-tau hyperphosphorylation by acting on GSK3β and also had neuroprotective effects and reduced ROS production [550]. It was demonstrated that this compound inhibits AChE by interacting mainly with its active center [535]. The antioxidant activity of morin has been shown to be associated with its effects on NF-κB, thereby modulating the ERK and p38 MAPKs pathways [551]. Furthermore, this compound in PD models showed neuroprotective effects both in vivo and in vitro [552,553]. It reduced apoptosis in PCl2 cells and ROS formation. Morin alleviated behavioral deficits as well as nigrostriatal damage in mice by ameliorating dopamine loss. In these animals, motor dysfunction was reduced after morin administration. Morin also showed beneficial effects in ameliorating depressive symptoms in rats exposed to chronic mild stress [554]. Alleviation of depressive symptoms was observed in SPT, OFT and FST. Animals in the hippocampus showed increased levels of serotonin, epinephrine and norepinephrine and decreased levels of glutathione and malondialdehyde. It also acted on the apoptosis pathway through caspase-3. In addition, it was shown to play a role in the inflammasome theory, which has a role in explaining the pathogenesis and progression of MDD. Morin also decreased the levels of TLR-4, TNF-α and IL-1β, as well as caspase-1.

α-Mangostin is also a compound that belongs to the xanthones group. It is a compound found mainly in Garcinia mangostana. It was shown that it decreased βA production by inhibiting β-secretase and γ-secretase [555]. However, it did not affect the expression of genes involved in the amyloidogenic and non-amyloidogenic pathway and APP maturation in cell cultures. This compound could potentially bind to PS1 and BACE1. In other studies, derivatives of this compound in a rat model of AD ameliorated neurological and behavioral disorders [556]. It was observed to improve cognitive function and reduce neuronal damage. In rats, there were improvements in memory and learning after administration of derivatives of this compound. In addition, they reduced the accumulation of Aβ1-42 in the hippocampus in AD rats. In another study, aqueous extracts of Garcinia mangostana in scopolamine-treated rats showed similar effects as previously mentioned [557,558]. In addition to this, they had anti-apoptotic and antioxidant effects, as well as inhibitory effects on AChE. An extract dose of 100 μg/mL showed a maximum inhibitory effect on the enzyme of 50%. In addition to these effects, the compound also reduced p-tau levels. It had anti-inflammatory and antioxidant effects, as well as cytoprotective effects [559]. Additionally, it increased the levels of BDNF. Another study reported that the compound inhibited nervous system inflammation mediated by microglia. In addition, it also acted on the TAK1/NF-κB pathway [560]. Similar to the previously mentioned studies, the compound showed neuroprotective and antioxidant effects and reduced memory and learning deficits. α-Mangostin has also been tried for the treatment of PD [561]. In cell cultures, the compound had an inhibitory effect on glial neuroinflammation and α-synuclein-induced neurotoxicity. In addition, it reduced the levels of pro-inflammatory cytokines IL-6 and TNF-α, and had antioxidant effects. It inhibited microglia activation, thus exhibiting neuroprotective effects. Moreover, to these effects, inhibition of caspase-3 and -8 activation was also observed after treatment of cells with this compound, as well as reduced mitochondrial dysfunction [562]. It also decreased α-synuclein accumulation, thereby protecting dopaminergic neurons. Furthermore, α-mangostin treatment in rotenone-treated animals showed restoration of locomotor performance, reduced memory deficits and affected levels of oxidative stress enzymes [563]. It was also mentioned that it has anti-inflammatory activity. This compound was shown to reduce the levels of IL-6 and cyclooxygenase-2 [564]. However, it did not statistically significantly reduce the levels of IL-1β and TNF-α. Noteworthy, α-mangostin showed a protective effect on neurons against 3-NPA toxicity [565]. In the same study, it showed its ability to scavenge ROS. In addition, α-mangostin has also been shown to have antidepressant effects. This effect is due to the fact that this compound acts on dopaminergic, serotonergic and glutaminergic systems [566]. Another isolated compound from *Garcinia mangostana* is Tovophyllsin A [567]. This compound exerted protective effects on dopaminergic neurons in PD and also reduced behavioral dysfunction in animals. The researchers concluded that the neuroprotection for this compound may be due to its effects on the Akt/GSK-3β pathway.

## 7. Conclusions

AChE plays an important role in the neurodegenerative diseases. Understanding its role will allow even better comprehension of the pathogenesis and pathophysiology of these disorders. This enzyme has several important functions that are common to most of the described disorders, i.e., participation in oxidative stress and inflammatory response, role in apoptosis and role in adhesion of pathological proteins. AChE is an enzyme that degrades one of the most important neurotransmitters, ACh. Disturbances of its levels may contribute to neurodegenerative diseases as well as depressive disorders. In addition to inhibiting the enzyme, AChE inhibitors currently on the market have a number of other properties that may help slow disease progression. It is commonly believed that they act only symptomatically and not causally. This review does not quite live up to that theory, since some of them may have potential use in causal treatment. AChE has a characteristically constructed active center and PAS. Many MTDLs currently being designed target AChE. More modelling and substrate docking studies would allow us to find a compound that will be an excellent AChE inhibitor, with the fewest side effects and with good penetration across the blood–brain barrier. The design and search for new drugs targeting AChE may in the future allow for the discovery of a therapies that will be effective in more neurodegenerative diseases, due to the fact that this enzyme plays a significant role in most of them.

The use of AChE inhibitors in the treatment of depressive disorders such as MDD, LOD or BPAD has many limitations. The use of these inhibitors should be considered in the absence of other therapeutic options or as adjunctive treatment. However, for this to be possible, more research should be conducted in this direction. In addition, studies should be directed towards understanding the cause of the differential response to AChE inhibitors in depressive disorders.

The problem with the use of plant extracts in the treatment of these diseases is their phytochemical variability. It causes inconsistency in results of individual extracts due to different potency of AChE inhibition and different antioxidant or anti-inflammatory properties. It is important to find promising results of a given extract, isolate its components and search for the compounds responsible for the obtained results. Then, they should be modified in such a way to obtain the best pharmacodynamic and pharmacokinetic properties.

## Figures and Tables

**Table 1:** Characteristics of the mentioned drugs.

Drug	Characteristics	Disadvantages
**Well-known inhibitors of AChE**		
Rivastigmine	Inhibitor of AChE and BChE Drug registered for the symptomatic treatment of mild to intermediate dementia in AD and idiopathic PD Administered as an oral or transdermal patch Effects on βA levels Beneficial effects in AD and PD Anti-inflammatory effects	Side effects (mainly gastrointestinal) Oral administration requires dose control Discrepant study results regarding use in HD, MDD, BPAD, PSP No proven efficacy in SM No efficacy data in ALS, OPCA
Donepezil	Drug registered for the symptomatic treatment of mild, moderate and severe dementia in AD AChE inhibitor Has almost no drug interactions May be a matrix-type drug Improves the condition of patients with AD, PD Alleviates cholinergic deficits in some part Exhibits effects on βA, p-tau	Side effects (similar to rivastigmine) Gene polymorphisms affect response to the drug Divergent findings regarding use of the drug in SM, HD, BPAD, MDD, PSP No data available for ALS, OPCA
Galantamine	AChE inhibitor Registered as a drug in patients with mild to moderate forms of AD Large volume of distribution May be a prolonged-acting or immediate-acting drug Improves cognitive abilities in AD Anti-inflammatory, antioxidant activity -Effects on βA Beneficial effects on symptoms associated with HD	Side effects as above Discrepant findings regarding efficacy in PD, BPAD, MDD No data available for ALS, SM, PSP, OPCA
Huperzine A	It is not yet registered as a drug in any neurodegenerative disease (Ex. China) Mild to moderate side effects Neuroprotective effects Beneficial effects in patients with AD Effects on βA	No data available for other neurodegenerative diseases
Phenserine Posiphen	AChE inhibitor Effects: Anti-inflammatory Antioxidant Neuroprotective Anti-apoptotic Beneficial effects in PD Promising results in PD and AD in animals	Phenserine failed in clinical trials No data available for other neurodegenerative diseases
**New compounds with potential use in the treatment of neurodegenerative diseases and depression**		
Thunberginol C	Inhibitors of AChE and BChE Anti-inflammatory activity (?)	A small amount of scientific research
Hydrangenol 8-O-glucoside pentaacetate		
Hydrangenol	Effects: Anti-inflammatory Antioxidant Anti-apoptotic	No data available on effects on AChE A small amount of scientific research
Skimmin	Effects: Anti-inflammatory Antioxidant (?) Cytoprotective (?)	
Extracts and essential oils of *Salvia* spp.	Most promising compounds: Salvianolic Acid B Tanshinone I tanshinone IIA Fruticuline A Caffeic acid Beneficial for cognitive abilities and memory improvement Effects on mood Effects on p-tau, βA, HTT, α-synuclein Effects on microglia Inhibitors of AChE Effects Antioxidant Anti-inflammatory Antidepressant Antinociceptive Neuroprotective	The chemical profile of an oil/extract depends on various climatic, soil or latitude and longitude factors
Extracts and essential oils of *Prunus* spp.	Most promising compounds: Chlorogenic acid Phytol Effect: Antioxidant Anti-inflammatory Neuroprotective Antinociceptive Beneficial effects on cognitive abilities AChE inhibitors	
Hesperidin	Effects on βA, p-tau Inhibitor of AChE and BChE Effects on cognitive abilities (better than donepezil) Promising results in AD, PD, HD, SM, ALS, MDD, BPAD Effects: Anti-inflammatory Anti-apoptotic Neuroprotective	
Limonoids	Effects on βA, AChE Effects on cognitive abilities (better than donepezil) Neuroprotective effect	
Naringenin	AChE Inhbitiror Effects on βA, Beneficial effects on cognitive abilities Promising results in PD, AD, MDD, HD, BPAD, ALS, SM Effects: Antioxidant Neuroprotective Anti-apoptotic Antidepressant Antinociceptive	
Hesperetin	Effect Cytoprotective Anti-inflammatory Anti-apoptotic Antioxidant Antidepressant	
Narirutin	Effects on βA Low toxicity Effects: Antioxidant Antidepressant	
Mangiferin	AChE inhibitor Effects on p-tau, βA (α-Mangostin) Potential use: AD, PD, MDD Effects: Cytoprotective Anti-inflammatory Antioxidant Antidepressant Antiapoptotic	
Morin		
α-Mangostin		

## Data Availability

Not applicable.
